# N-Acetyl Aspartic Acid (NAA) Attenuates Stemness and Epithelial–Mesenchymal Transition and Enhances Radio- and Chemo-Sensitivity in Pancreatic Ductal Adenocarcinoma

**DOI:** 10.3390/cells15171616

**Published:** 2026-09-05

**Authors:** Fabiana Crispo, Rosa Lioy, Rosalia Dieli, Mara Martinelli, Carlo Calabrese, Antonella Bianculli, Vincenzo De Fina, Grazia Lazzari, Vito Metallo, Donatella Telesca, Gennaro Laus, Simona Loperte, Rosa Lerose, Carmela Mazzoccoli

**Affiliations:** 1 Laboratory of Preclinical and Translational Research, IRCCS-CROB, Centro Di Riferimento Oncologico della Basilicata, 85028 Rionero in Vulture, PZ, Italy; rosa.lioy@crob.it (R.L.); rosalia.dieli@crob.it (R.D.); mara.martinelli@crob.it (M.M.); carlo.calabrese@crob.it (C.C.); 2 Physic Unit, IRCCS-CROB, Centro Di Riferimento Oncologico della Basilicata, 85028 Rionero in Vulture, PZ, Italy; antonella.bianculli@crob.it (A.B.); vincenzo.defina@crob.it (V.D.F.); 3 Radiation Oncology Unit, IRCCS-CROB, Centro Di Riferimento Oncologico della Basilicata, 85028 Rionero in Vulture, PZ, Italy; grazia.lazzari@crob.it (G.L.); vito.metallo@crob.it (V.M.); 4 Hospital Pharmacy, IRCCS-CROB, Centro Di Riferimento Oncologico della Basilicata, 85028 Rionero in Vulture, PZ, Italy; donatella.telesca@crob.it (D.T.); rosa.lerose@crob.it (R.L.); carmela.mazzoccoli@crob.it (C.M.); 5 Department of Services, IRCCS-CROB, Centro Di Riferimento Oncologico della Basilicata, 85028 Rionero in Vulture, PZ, Italy; gennaro.laus@crob.it; 6 Institute of Methodologies for Environmental Research, National Research Council of Italy, 85050 Tito Scalo, PZ, Italy; simona.loperte@cnr.it

**Keywords:** pancreatic ductal adenocarcinoma, N-acetylaspartic acid, epithelial–mesenchymal transition, stemness, drug resistance, radiosensitization

## Abstract

Pancreatic ductal adenocarcinoma (PDAC) is characterized by poor clinical outcomes and limited responsiveness to conventional treatments. Beyond delayed diagnosis, its high mortality is linked to limited treatment choices and resistance to chemotherapy. Neoplastic cells characterized by stem-like features and epithelial–mesenchymal transition (EMT) activation are crucial drivers of drug resistance and metastatic progression. Consequently, targeting these cellular programs could represent a promising therapeutic strategy for PDAC. N-acetyl-L-aspartic acid (NAA) is an endogenous metabolite, mainly localized in the central nervous system, where it facilitates acetate storage for lipid metabolism. Previous studies established its antineoplastic effect in neuroblastoma, promoting a more differentiated phenotype of cancer cells. Here we investigated the effects of NAA on BxPC-3 pancreatic cancer cells using both 2D and 3D models. NAA treatment induced a dose-dependent reduction in cell proliferation and led to a significant downregulation of stemness-associated surface markers, with concomitant upregulation of E-cadherin. Furthermore, NAA significantly enhanced cellular radiosensitization with a reduction in caveolin-1 and increased efficacy of gemcitabine in PDAC cells. Collectively, these results confirmed the anticancer activity of NAA and provide a rationale for further investigation of its synergistic effects with radiotherapy to yield better PDAC treatments.

## 1. Introduction

Pancreatic ductal adenocarcinoma (PDAC) is the most predominant histological subtype of pancreatic cancer, representing approximately 90% of all pancreatic tumors [1]. Due to the silent progression of the disease, lack of screening programs, and late-stage diagnosis, PDAC remains one of the most aggressive malignancies, with a five-year survival rate of around 10–13% [1,2]. Current projections indicate a worrying increase in the global burden, making PDAC one of the top causes of cancer-related deaths in the future [3,4].

Current treatment options for PDAC are limited, with surgical resection being the most effective for patients with localized disease and at an early stage [5]. However, only a small percentage of patients are eligible for surgery. For those with advanced disease or late-stage diagnosis, chemotherapy remains the primary treatment. Standard regimens include FOLFIRINOX or gemcitabine, either as monotherapy or in combination with nab-paclitaxel [6,7]. Despite these treatments, the overall prognosis for PDAC patients remains poor. Therefore, it is critical to intensify research into the molecular mechanisms driving the onset and progression of PDAC, aiming to discover novel therapeutic targets, as well as more effective compounds, to improve patient outcomes.

**Figure 1:**
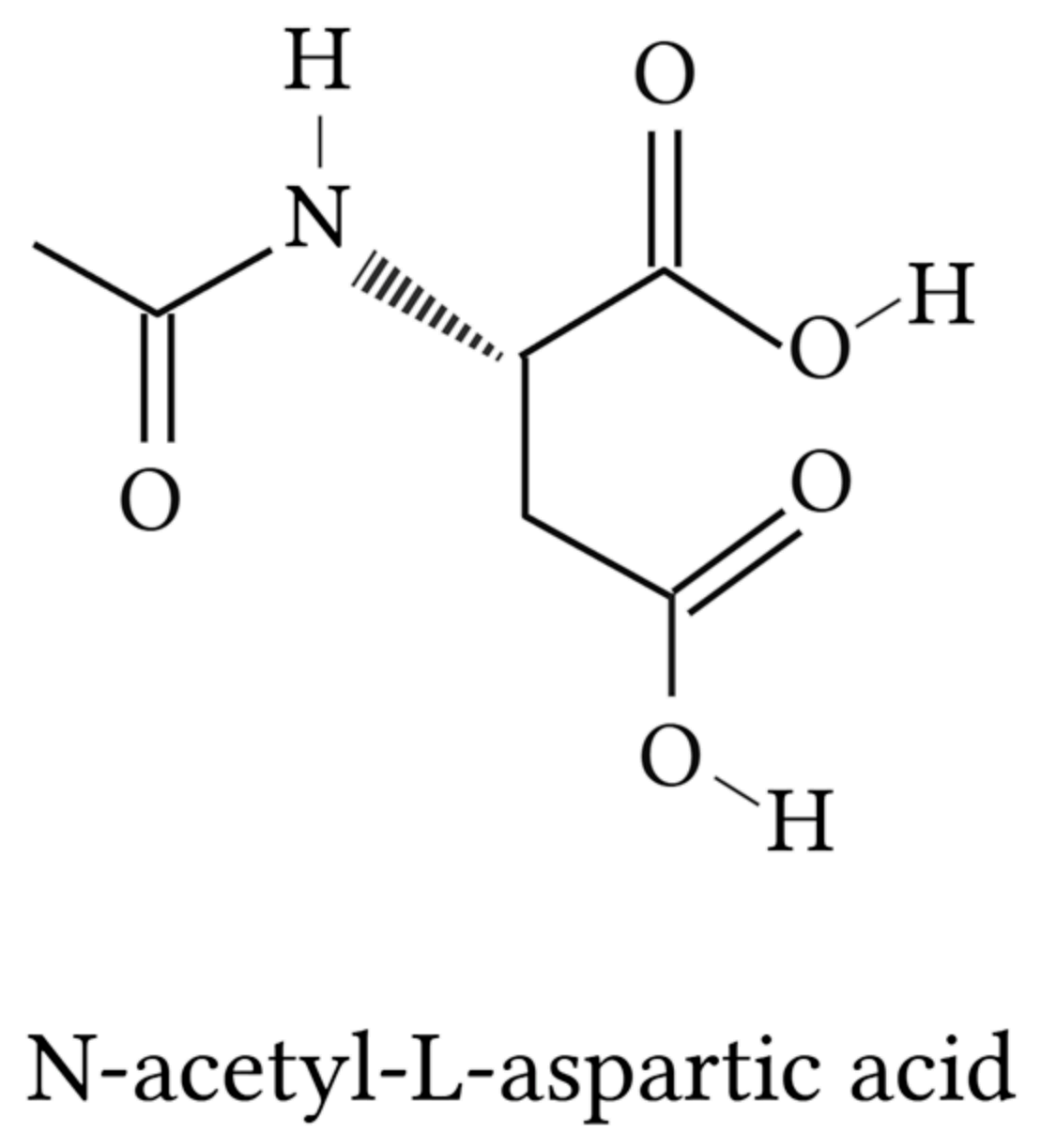
Chemical structure of N-acetyl-L-aspartic acid.

N-acetyl-L-aspartic acid (NAA) (Figure 1) is a nervous system-specific metabolite, synthesized by neurons through N-acetylation of L-aspartate (ASP), where acetyl-CoA is the acetyl group donor.

NAA is involved in myelin lipid synthesis during both brain development and its turnover in adults; NAA probably also takes part in other metabolic processes, such as a bioenergetic role in neuronal mitochondria [8,9]. Thus, defective NAA synthesis or catabolism may alter its availability and compromise neuronal metabolism, correlating with neuropathologic conditions [8,10]. Considering this, NAA is widely regarded as a biomarker for neuronal integrity and viability [11]. Although it is predominantly in the brain, NAA is also present in other organs [12,13,14,15], including the pancreas [16], although its physiological functions in non-neuronal tissues are largely unknown.

Metabolic rewiring is one of the hallmarks of cancer [17]. Its dynamic and reversible nature enables neoplastic cells to rapidly adapt to a hostile surrounding environment. Ancillary and secondary metabolic pathways are not free from this remodeling, and alterations in NAA metabolism may represent an alternative strategy by which cancer cells could support survival and rapid proliferation, particularly under nutrient-deprived or hypoglycemic conditions [15,18]. Cancer tissues exhibit marked variations in NAA levels, largely depending on tumor type and biological context. In ovarian or lung cancers, the concentration of NAA is elevated [19,20,21,22], whereas significant reduction has been reported in PDAC and hepatocellular carcinoma (HCC) [16,23]. Thus, whether NAA supports or counteracts tumor progression remains debated. Indeed, previously, our group explored the potential therapeutic effects of NAA on neuroblastoma cells, demonstrating its ability to promote in vitro neuronal differentiation and to increase the sensitivity to chemotherapy [24]. Independently, a pro-apoptotic effect of NAA treatment was demonstrated in glioblastoma cancer cells [25].

Given the promising efficacy of NAA in some CNS malignancies and considering its depletion in PDAC tissues, the present study investigated the anti-tumor effects of exogenous N-acetylaspartate on pancreatic cancer 2D and 3D models. For this purpose, BxPC-3 cells and their spheroids were used as models for experiments with NAA. The results indicate significant antiproliferative effects in pancreatic cancer models, particularly when combined with ionizing radiation and gemcitabine, the standard chemotherapeutic regimen for PDAC, positioning NAA as a viable candidate for integrated treatment strategies in pancreatic cancer.

## 2. Materials and Methods

### 2.1. Cell Culture for 2D and 3D Models

The human pancreatic adenocarcinoma cell lines BxPC-3 (CRL-1687) and PANC-1 were purchased from the American Type Culture Collection (ATCC, Manassas, VA, USA) and cultured at 37 °C in a 5% CO_2_ humidified atmosphere, routinely monitored by microscopic observation of cell morphology (Nikon Eclipse TE300, Nikon Inc., Tokyo, Japan). BxPC-3 cells were grown in complete RPMI 1640 (Roswell Park Memorial Institute Medium, Thermo Fisher Scientific, Waltham, MA, USA), supplemented with 10% fetal bovine serum (FBS), 1% penicillin–streptomycin (10.000 U/mL), and 1% L-glutamine 200 mM. PANC-1 cells were cultured in high-glucose DMEM (Dulbecco’s Modified Eagle Medium, 4.5 g/L) supplemented with 10% fetal bovine serum (FBS), 1% penicillin–streptomycin (10.000 U/mL), and 1% L-glutamine 200 mM. Unless otherwise specified, reagents were purchased from Thermo Fisher Scientific (Waltham, MA, USA).

Cells were treated with N-Acetyl-L-aspartic acid (NAA) at known concentrations for experimental purposes. N-Acetyl-L-aspartic acid was obtained from Sigma-Aldrich (#00920, St. Louis, MO, USA). Before use, NAA serial dilutions were prepared freshly in culture medium, at the concentrations required for the experiments, using a stock solution. The 160 mM stock solution was obtained by dissolving the free acid powder in sterile water for injection and adjusting the pH to 7.4 before bringing it to final volume. Finally, the stock solution was sterilized by filtration using a 0.22 μm filter. For each NAA concentration tested, changes in cell morphology were observed using an inverted optical microscope (Axio Vert A1, Zeiss, Oberkochen, Germany).

3D culturing of BxPC-3 was performed by seeding 1.0 × 10^3^ cells/well into ultra-low attachment 96-well round-bottomed plates, which were cultured in supplemented RPMI at 37 °C under 5% CO_2_ atmosphere for 7 days until spheroid formation. For the evaluation of the NAA effects on spheroid formation and/or growth, three different experimental protocols were followed: (i) NAA solutions at different concentrations (2, 4 and 8 mM) were added directly to the BxPC-3 cell suspension and seeded into ultra-low attachment plates, and the culture was maintained for 7 days; (ii) BxPC-3 cells were pretreated with 2, 4 and 8 mM of NAA for 72 h and then seeded into ultra-low attachment plates, maintaining the 3D culture in RPMI medium, NAA-free, for 7 days; (iii) untreated BxPC-3 cells were maintained in standard culture conditions for 7 days until spheroids formed and were then treated with NAA at 2, 4 and 8 mM concentrations for 72 h.

Spheroids of PANC-1 were generated by seeding 1.0 × 10^3^ cells/well into ultra-low attachment 96-well round-bottomed plates in supplemented high-glucose DMEM at 37 °C under 5% CO_2_ atmosphere for 7 days, until their formation. The pre-formed spheroids were then treated with NAA at 2, 4 and 8 mM concentrations for 72 h.

Spheroid diameter was measured using the ZEISS ZEN imaging software based on pictures obtained by an inverted optical microscope (Axio Vert A1, Zeiss, Oberkochen, Germany). Spheroid viability was evaluated using the MTS (Methan Thiazolyl Tetrazolium) test (CellTiter 96 Aqueous One Shot Assay #G3580, Promega, WI, USA). The absorbance values were measured at a wavelength of 490 nm using a VICTOR Nivo Multimode Microplate Reader (PerkinElmer, Life and Analytical Sciences, Waltham, MA, USA). The obtained absorbance data were analyzed using the GraphPad Prism 11.0 software, and IC50 values were calculated through non-linear regression analysis by creating a dose–response curve. All experiments were performed in triplicate.

### 2.2. Real-Time Cell Proliferation and Migration Monitoring by xCELLigence System

The effects of NAA treatment on cell proliferation were evaluated by a real-time cytotoxicity assay [26,27]. The xCELLigence experiments were performed using the RTCA (Real-Time Cell Analyzer) system according to the manufacturer’s instructions (ACEA Biosciences, San Diego, CA, USA). The optimal cell seeding density was preliminarily determined through cell titration and growth curve experiments. BxPC-3 cells were seeded into E-plate 16 (#5469830001, Agilent, Santa Clara, CA, USA) with 2.5 × 10^3^ cells/well, and cell proliferation was automatically monitored every 30 min following the usual protocol. After an overnight incubation to allow for adhesion to the bottoms of the plate wells, cells were treated with increasing concentrations of NAA (2 mM, 4 mM, 8 mM and 16 mM) or 2.5 µM gemcitabine. The cell index (CI) was continuously recorded for up to 90 h after seeding. Data were analyzed using the xCELLigence software (Version 2.0, ACEA Biosciences, San Diego, CA, USA) and expressed as the mean ± SD of the normalized CI, calculated relative to the last cell index value recorded before NAA treatment, (Agilent Technologies Inc, Santa Clara, CA, USA.) were used to evaluate the effects of NAA treatment on BxPC-3 cell migration. NAA-treated cells were seeded in serum-free RPMI medium in the upper chamber of a CIM Plate at an optimal 3 × 10^3^ cells/well. With 10% FBS-supplemented DMEM in the lower chamber, cells received the chemotactic signal for transwell migration through the 8 µm pores of the PET membrane between the upper and lower chambers. CI was monitored every 15 min for up to 24 h, and curves show the average of the wells in triplicate.

### 2.3. Clonogenic Assay

For the clonogenic assay, a cell suspension of 500 cells was seeded in each well of 6-well plates and cultured in complete RPMI medium. After attachment, BxPC-3 cells were treated with different NAA concentrations, and cultures were incubated at 37 °C in a fully humidified atmosphere supplemented with 5% CO_2_. After 72 h of NAA treatment, the colonies were fixed with 4% paraformaldehyde at room temperature (RT) for 10 min. After the fixation solution was removed, colonies were stained with crystal violet solution for 20 min, rinsed with H_2_O, and air-dried at RT. Colonies were counted under a light microscope and quantified using a 500 µm diameter threshold value, as previously reported [28]. Each assay was performed in triplicate.

### 2.4. Cell Cycle Analysis and Apoptosis Assay with Flow Cytometer

Cell cycle analysis experiments were performed as previously reported [29]. For cell cycle experiments, BxPC-3 cells were seeded in 6-well plates at a density of 1.0 × 10^5^ cells/well and cultured in RPMI complete medium. After 24 h to allow for cell adhesion, cells were treated with NAA at a concentration range of 2 mM to 8 mM for 72 h before sample preparation for cell cycle analysis. The flow cytometric analysis was carried out with the DxFlex flow cytometer (Beckman Coulter, Brea, CA, USA). A total of 1.0 × 10^4^ events were acquired and analyzed for cell cycle analysis. The cell cycle results were expressed as a percentage of cells in G0/G1, S, and G2/M phases (mean ± SEM of three independent experiments), and the cell cycle distribution was analyzed using the Kaluza Analysis 1.3 software (Beckman Coulter). For apoptosis evaluation after NAA treatment, BxPC-3 cells were stained with Annexin-V-FITC and PI using the FITC Annexin V Apoptosis Detection Kit (Becton Dickinson, Franklin Lakes, NJ, USA). For each sample, 1.0 × 10^4^ events were acquired to detect live, apoptotic and necrotic cells. Three independent experiments were carried out for each cytometric assay.

### 2.5. Cytofluorimetric Detection of Cell Stemness Surface Markers

Evaluation of CD133 and CD184 surface marker expression in BxPC-3 cells was performed via cytofluorimetric analysis [30]. For the staining, cell suspensions were reconstituted to a final concentration of 1.0 × 10^6^ cells/mL in phosphate-buffered saline (PBS) containing 0.1% (*w*/*v*) NaN3 and 5% (*v*/*v*) FBS. For specific staining, 100 μL of each cell suspension was incubated with 10 μL of each primary antibody for 20 min at 4 °C in the dark. The following primary antibodies were used: mouse anti-human R-phycoerythrin (PE) CD133/2 antibody (1:10; #130-113-186; Miltenyi Biotec, Bergisch Gladbach, Germany) and mouse anti-human allophycocyanin (APC) CD184 antibody (1:50; #560936; BD Pharmigen, Franklin Lakes, NJ, USA). After washing with PBS, cells were resuspended in PBS and were finally analyzed by a DxFlex flow cytometer. Initial gating was established based on physical parameters (FSC-A vs. SSC-A) to exclude debris. To define marker-positive populations, cells were stained with the corresponding isotype controls, specifically IgG2Bκ PE (#130-092-215; Miltenyi Biotec, Bergisch Gladbach, Germany) and IgG2Aκ APC (#555576; BD Pharmigen, NJ, USA) for CD133 and CD184 respectively. Positivity thresholds were determined using a histogram of the isotype controls. The data analysis was performed using the Kaluza Analysis 1.3 software (Beckman Coulter).

### 2.6. Real-Time RT-PCR Analysis

Total RNA was extracted from cell pellets using the TRIzol Reagent (Life Technologies, Carlsbad, CA, USA). The RNA concentration and purity were assessed using NanoDrop technology (Thermo Fisher Scientific, Waltham, MA, USA), considering absorbance at 260 nm and a 260/280 nm absorbance ratio respectively. For first-strand synthesis of cDNA, 1 μg of RNA was used in a 20 μL reaction mixture utilizing a Transcriptor First Strand cDNA Synthesis Kit (Roche, Mannheim, Germany). For real-time PCR analysis, 0.5 ng of the cDNA samples was amplified using the LightCycler 480 SYBR Green I Master (Roche) in a LightCycler 480 (Roche). The sequences of primers TP53, CDKN1A and CDKN1B were purchased from Sigma Aldrich and are reported in Table 1. For stemness genes OCT4 (POU5F1, #QT00210840), KLF4 (#QT00061033) and c-MYC (#QT00035406), a mix of lyophilized forward and reverse primers (QuantiTect Primer Assay, Qiagen, Hilden, Germany) was used, according to the manufacturer’s protocol.

The reaction conditions were as follows: pre-incubation at 95 °C for 5 min, followed by 50 cycles of denaturation at 95 °C for 10 s, annealing at 59 °C for 10 s, and extension at 72 °C for 10 s. The relative amounts of target genes were normalized to GAPDH expression using LightCycler^®^ 480 Software version 1.5 (Roche Diagnostics) and calculated by the 2^−ΔΔCt^ method.

### 2.7. Immunoblot Analysis

Total cell lysates of BxPC3 cells, treated with 4 mM and 8 mM NAA, were obtained by homogenizing cell pellets for 30 min in a cold lysis buffer, made of 20 mM Tris, pH 7.5, containing 300 mM sucrose, 60 mM KCl, 15 mM NaCl, 5% (*v*/*v*) glycerol, 2 mM EDTA, 1% (*v*/*v*) Triton X-100, 1 mM PMSF, 2 mg/mL aprotinin, 2 mg/mL leupetin, and 0.2% (*w*/*v*) deoxycholate, with 1X halt protease and phosphatase inhibitor cocktails (#78429 and #1861277, Thermo Fisher Scientific, Waltham, MA, USA) [30]. Immunoblot analysis of 40 μg of protein was performed as previously reported [31]. The following primary antibodies, conveniently diluted as reported in the datasheet, were used: E-Cadherin (#610181, BD Biosciences, San Jose, CA, USA, 1:1000) and β-actin (A5441, Sigma Aldrich, Milano, Italy, 1:2000). After 1 h of incubation with the correspondingly suited horseradish peroxidase-conjugated secondary antibody (goat anti-mouse IgG (H + L)-HRP-conjugated #170-6516; goat anti-rabbit IgG (H + L)-HRP-conjugated #170-6515; Bio-rad, Hercules, CA, USA, 1:2000), signals were developed using an enhanced chemiluminescence kit (Clarity™ Western ECL Substrate, Bio-Rad Laboratories Inc., Hercules, CA, USA) and detected using the chemiluminescence ChemiDoc Imaging System XRS (BioRad Laboratories GmbH, Hercules, CA, USA).

### 2.8. Experimental Radiation

Cell irradiation was performed using a Varian 2100 DHX linear accelerator (Varian Medical Systems, Palo Alto, CA, USA). After adhesion, cells were exposed to prescribed doses of 2, 4, or 6 Gy using a 6 MV photon beam delivered at a dose rate of 300 MU/min. Irradiations were performed with a 20 × 20 cm^2^ field size. Culture dishes were positioned at a source-to-surface distance (SSD) of 100 cm and aligned with the beam central axis to ensure homogeneous dose distribution across the irradiated area. The prescribed doses of 2, 4, and 6 Gy corresponded to 200, 400, and 600 monitor units (MU). All irradiations were delivered in a single fraction under identical experimental conditions. Dosimetric parameters were verified according to the institutional quality assurance program, and accelerator calibration was performed following the International Atomic Energy Agency (IAEA) TRS-398 protocol.

### 2.9. Confocal Microscopy Analysis

For immunofluorescence experiments, about 3.0 × 10^4^ cells were seeded into 4-well chamber slides (Cell Culture Slide I, SPL Life Sciences Co., Ltd., Pocheon-si, Gyeonggi-do, Republic of Korea) and cultured at 37 °C for adhesion. The day after, cells were treated with 8 mM NAA. After 72 h of NAA treatment, cells were irradiated at 4 Gy, and fluorescence was analyzed after 24 h of irradiation. BxPC-3 cells were washed with PBS and fixed with 0.1 M PBS containing 4% (*v*/*v*) formaldehyde (#F8775; Sigma-Aldrich, Saint Louis, MO, USA) for 15 min at room temperature (RT). Then, cells were permeabilized with 0.1% (*v*/*v*) and blocked with a blocking buffer (5% BSA (*w*/*v*) in PBS) for 30 min at room temperature before staining with primary antibodies. Finally, the chambers were incubated overnight at 4 °C with 5% BSA (in PBS) containing the following primary antibody: p21 Waf1/Cip1 (12D1) rabbit monoclonal antibody (#2947, Cell Signaling Technology, Danvers, MA, USA; 1:100); pH2AX (Ser139) mouse monoclonal antibody (#ab22551, Abcam, Cambridge, UK, 1:100) and Caveoli-1 (#3238, Cell Signaling Technology, 1:100). After washing steps with PBS, an incubation with Alexa-Fluor 488 goat anti-rabbit IgG secondary antibody (#A-11070, Thermo Fisher Scientific) and Alexa-Fluor 647 goat anti-mouse IgG secondary antibody (#A-21235, Thermo Fisher Scientific), 1:1000, in PBS with 5% BSA was carried out for 1 h at RT in the dark. Cell nuclei were counterstained with 4′,6-diamidino-2-phenylindole (DAPI, Thermo Fisher Scientific) as follows: after three washes with PBS, the slides were mounted using ProLong™ Glass Antifade Mountant with NucBlue™ (Thermo Fisher Scientific) for cell nucleus counterstaining. Images were acquired using a Leica SP8 confocal microscope (Leica Microsystems GmbH, Wetzlar, Germany), exciting the stained cells with laser light.

### 2.10. Statistical Analysis

Data are presented as means ± Standard Deviation (SD) or Standard Error of the Mean (SEM). Statistically significant differences (*p* < 0.05) were assessed using an unpaired Student’s *t*-test, one-way ANOVA, or two-way ANOVA, as appropriate, with the GraphPad Prism software (version 11.0.2; GraphPad Software, Boston, MA, USA). Differences were considered statistically significant when *p* < 0.05.

## 3. Results

### 3.1. Growth Inhibitory Effects of NAA in Pancreatic Cancer Cells

**Figure 2:**
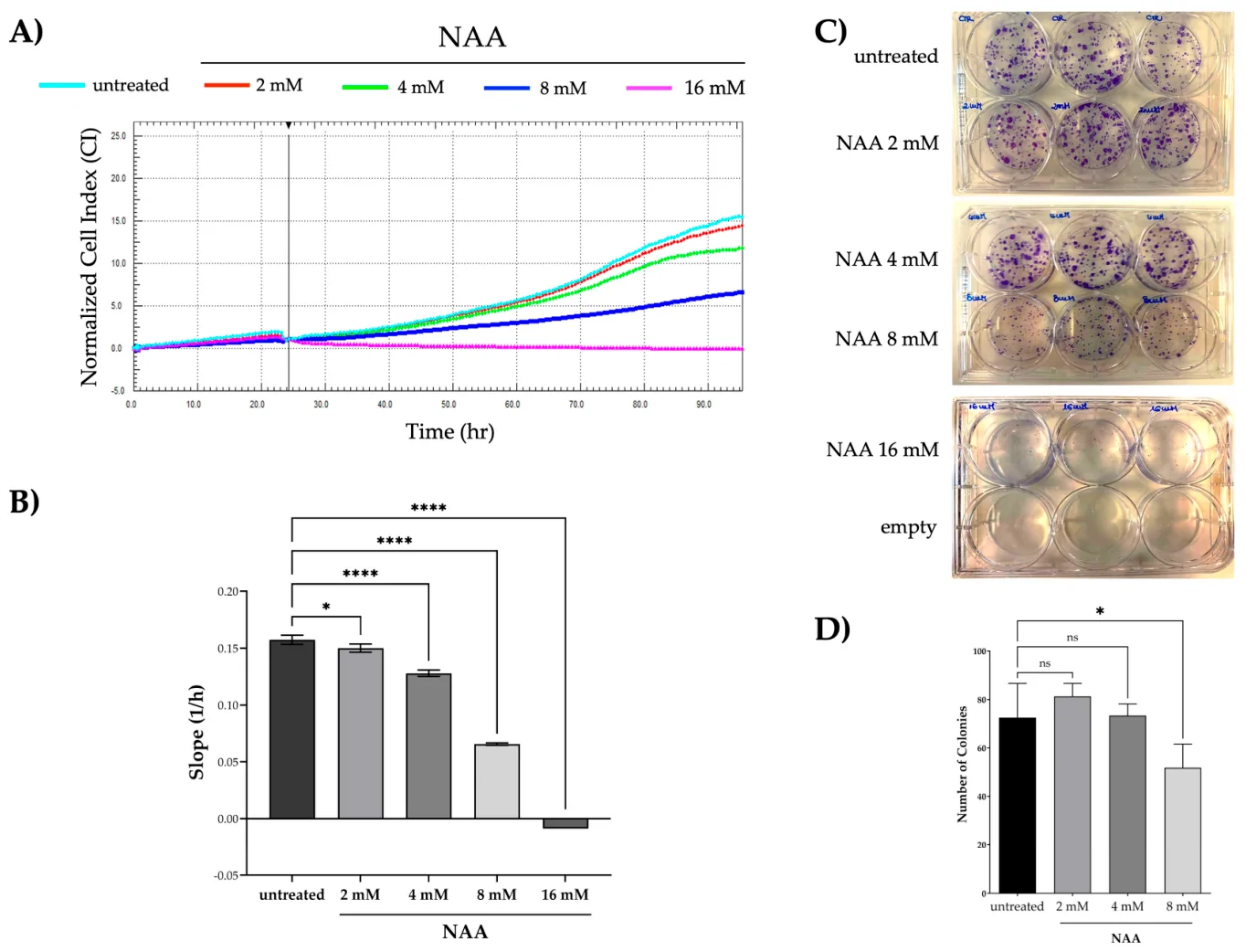
Antiproliferative and anti-clonogenic effects of NAA on BxPC-3. (**A**) Representative proliferation profile of BxPC-3 cells after 72 h of treatment with NAA at different concentrations (2–16 mM range), assessed using the xCELLigence system. Cell index (CI) values were normalized to the last measurement recorded immediately before NAA addition. (**B**) Proliferation rate of BxPC-3 cells treated with different concentrations of NAA, defined by the slope of the normalized CI curves during the 72 h treatment window. Bars represent the mean slope ± SD of three experiments. Statistical significance is shown for the indicated comparisons and was determined by one-way ANOVA; * *p* ≤ 0.05; **** *p* ≤ 0.0001. (**C**) Representative images of colony formation assays performed using untreated BxPC-3 cells or cells treated with increasing concentrations of NAA. In the wells of the empty line, no cells were seeded. (**D**) Bar plot of evaluation for colony formation after 72 h of NAA treatment. Bars represent the mean slope ± SD from three independent experiments. Statistical significance is shown for the indicated comparisons and was determined by one-way ANOVA; * *p* ≤ 0.05, ns (not significant).

Functional assays were performed in a BxPC-3 pancreatic cancer cell line to explore the anti-tumorigenic properties of NAA. The xCELLigence real-time system was used to assess the potential effect of NAA-induced inhibition on BxPC-3 cell growth. Treatment with NAA, at concentrations ranging from 2 to 16 mM for 72 h, inhibited cell proliferation in a dose-dependent manner, as demonstrated by the progressive decrease in normalized cell index (CI) values (Figure 2A). The antiproliferative effect of NAA was further quantified by slope analysis, which reflects the rate of change in CI over a defined time interval. A statistically significant reduction in proliferation rate was observed even at the lowest NAA concentration tested. At 8 mM, the proliferation rate was reduced by approximately 2.4-fold compared with untreated cells. At the highest concentration, 16 mM, the slope became negative, indicating a net decrease in CI that may be consistent with loss of cell viability and/or cell detachment (Figure 2B).

Subsequently, colony formation assays were performed to evaluate the long-term antiproliferative effects of NAA. Clonogenic capacity is a hallmark of neoplastic cells. Importantly, the colony-forming ability of these pancreatic cancer cells was markedly inhibited by the higher concentration of NAA, compared with the untreated control (Figure 2C). The count of colonies confirmed that a high dose of NAA impaired colony formation, with significantly smaller cell islands in the 8 mM condition. Notably, the size of the colonies observed in the wells treated with the highest concentration of NAA (16 mM) was below the detection threshold for counting; thus, no data were plotted for this condition (Figure 2D).

Proliferation assay experiments using xCELLigence were also performed on PANC-1 cells, with the aim of evaluating the antiproliferative effect of NAA on another pancreatic adenocarcinoma line. Interestingly, the 72 h NAA treatment reduced the proliferation rate of PANC-1 cells starting at 4 mM, with a potential cytotoxic effect at the highest NAA concentration (Supplementary Figure S1A,B), similar to that observed in BxPC-3 cells. Indeed, the negative slope value at 16 mM suggested a loss of adherent cells from the gold surface, which is compatible with cell death.

Taken together, these findings demonstrate that the antiproliferative effect of NAA is shared across another pancreatic cancer cell line. Moreover, the near-complete absence of cells following treatment with 16 mM NAA confirmed the cytotoxicity of this concentration, supporting the selection of 8 mM as the maximum dose of NAA for subsequent experiments, with BxPC-3 selected as the primary model for further deep investigations of the downstream biological effects.

### 3.2. NAA Regulates Cell Cycle Progression of BxPC-3 Cells

**Figure 3:**
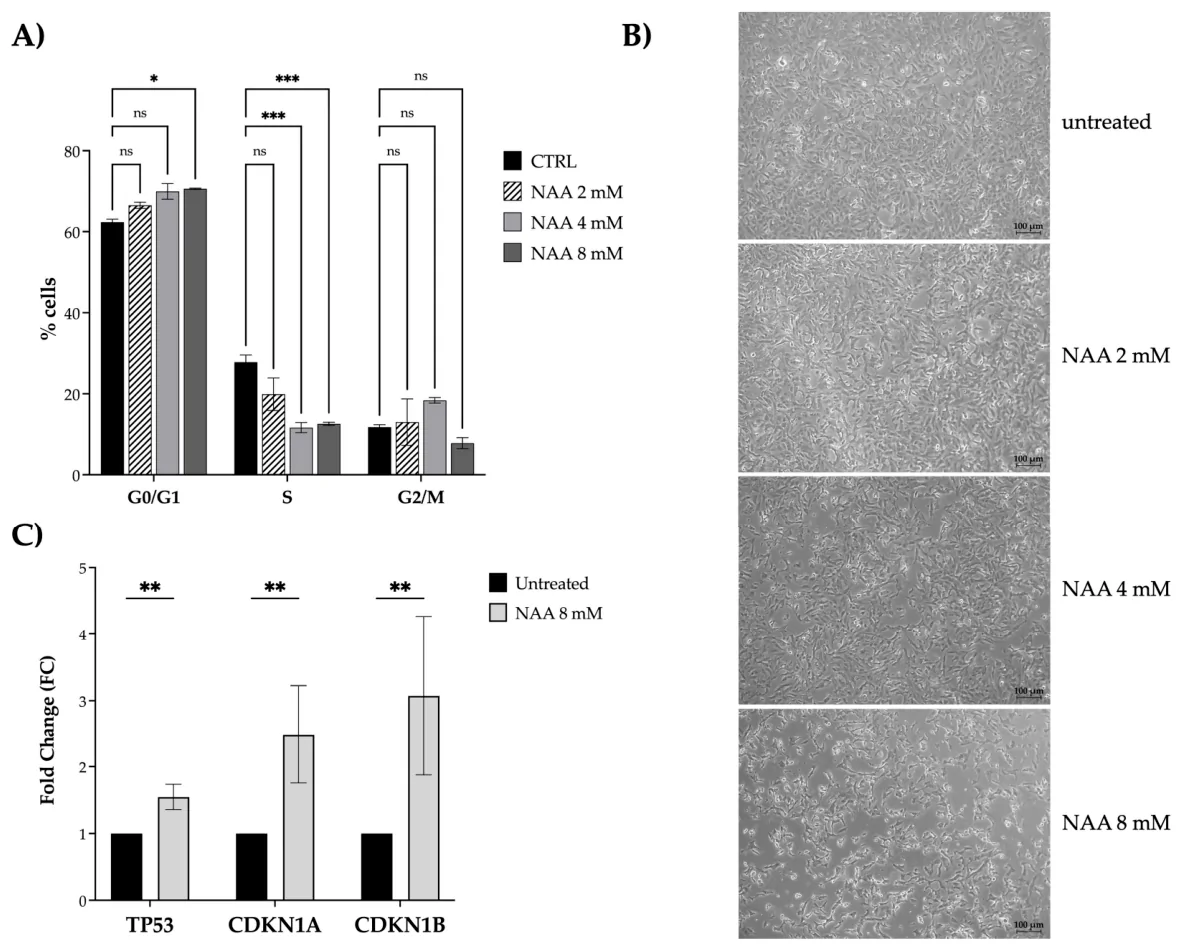
NAA affects the proliferation of BxPC-3 cells by acting on cell cycle progression. (**A**) Cell cycle distribution of BxPC-3 cells treated with 2, 4, or 8 mM NAA for 72 h, compared with untreated cells. The percentages of cells in the G0/G1, S, and G2/M phases were determined by flow cytometric analysis. The bar graph represents the mean ± SEM of three independent experiments. Statistical significance is shown for the indicated comparisons and was determined by two-way ANOVA: ns—not significant; * *p* ≤ 0.05; *** *p* ≤ 0.001. (**B**) Morphological analysis of BxPC-3 cells treated with increasing concentrations of NAA (ranging from 2 mM to 8 mM) compared to untreated cells. Representative phase-contrast microscopy images were acquired using a microscope at 20× magnification. Scale bars, 100 μm. (**C**) Relative expression levels of the cell cycle regulatory genes TP53 (p53), CDKN1A (p21), and CDKN1B (p27) in untreated cells and cells treated with 8 mM NAA. Expression values are presented as fold change (FC) relative to untreated cells. Data are shown as the mean ± SD from three independent experiments. Statistical significance was determined by unpaired *t*-test; ** *p* ≤ 0.01.

Since cell cycle regulation represents a primary mechanism of action for numerous therapeutic agents, we investigated the impact of NAA treatment on cell cycle progression in pancreatic cancer cells. Following 72 h of treatment, cell cycle distribution analysis revealed that NAA induced G1-phase arrest, as quantitatively demonstrated by flow cytometry histograms (Figure 3A and Supplementary Figure S2). In particular, at a concentration of 8 mM, a statistically significant effect of NAA on proliferation was observed, accompanied by an increase in the percentage of accumulated cells in the G0/G1 phase (70.0%) and a substantial decrease in cells in the S phase, which dropped to 12.6% compared to the 27.2% untreated cell population (Figure 3A).

This antiproliferative effect of NAA on BxPC-3 was also evident when comparing the microscope images of cells treated with increasing concentrations of NAA with untreated cells (Figure 3B), where the lower density of attached cells was evident. However, no evidence of an increase in detached cells was observed, and as expected, the cytometric apoptotic assay did not reveal a cytotoxic effect of NAA treatment, confirming that it has a more cytostatic than cytotoxic effect on BxPC-3-treated cells.

To elucidate the molecular mechanisms underlying the NAA-induced G1/S-phase arrest, the expression of key genes involved in G1 checkpoint regulation was evaluated. qPCR analysis showed that the cyclin-dependent kinase inhibitors p21 (CDKN1A) and p27 (CDKN1B) were significantly upregulated following the 8 mM NAA treatment (Figure 3C). These results supported the inhibitory effect of NAA on cell cycle progression and proliferation. TP53 expression also increased significantly, although to a lesser extent than that of p21 and p27.

Together, these observations suggest that NAA modulates G1/S-phase arrest through the activation of cell cycle inhibitory pathways at the transcriptional level.

### 3.3. NAA Reduces Migration Capability and Epithelial-to-Mesenchymal Transition (EMT) of BxPC-3 Cells

**Figure 4:**
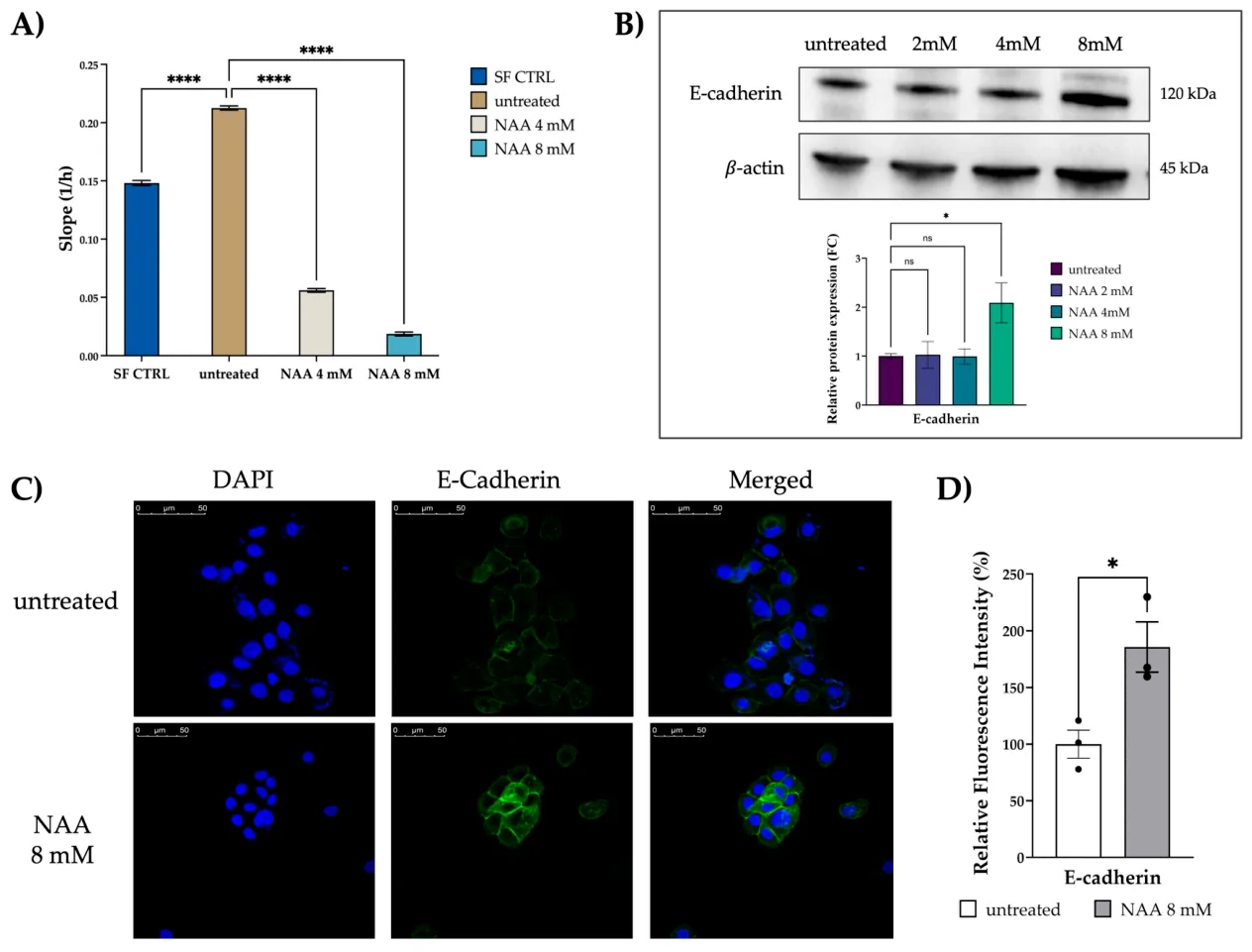
Effect of NAA on migration and E-cadherin expression. (**A**) The slope in SF CTRL, untreated cells, and cells treated with NAA at 4 or 8 mM. Bars represent the mean ± SD from three experiments. Statistical significance is shown for the indicated comparisons and was determined by two-way ANOVA: **** *p* ≤ 0.0001. (**B**) Representative Western blot showing E-cadherin protein levels in untreated cells and cells treated with 2 mM, 4 mM or 8 mM NAA for 72 h. The bar graph below the immunoblot represents the relative protein expression (means ± SD) obtained from densitometric analysis of three independent experiments. Statistical significance was determined by one-way ANOVA: ns—not significant; * *p* ≤ 0.05. (**C**) Representative immunofluorescence images of untreated cells and cells treated with 8 mM NAA for 72 h. Nuclei were stained with DAPI (blue), and E-cadherin (green) was detected in FITC. Merged images are shown in the right column. Scale bars, 50 μm. (**D**) Bar graph representing the percentage of E-cadherin fluorescence (mean ± SD), normalized with respect to the number of nuclei, across three distinct acquisition fields. Statistical significance was evaluated by unpaired *t*-test; * *p* ≤ 0.05.

The BxPC-3 cells’ migration abilities after NAA treatment were detected by the xCELLigence migration assay. The migration assay (Supplementary Figure S3) showed that NAA treatment significantly reduced the cell index of BxPC-3 cells, suggesting that NAA delays migration, potentially by regulating the epithelial–mesenchymal transition. Specifically, treatment with 4 mM and 8 mM NAA reduced the migration slope below that of the negative control (SF-CTRL) (Figure 4A). In contrast, untreated BxPC-3 cells migrated efficiently toward the chemoattractant, showing a steep slope well above the SF-CTRL. Together, these results demonstrate that NAA inhibits BxPC-3 cell migration in a dose-dependent manner.

EMT is a pathological process involved in the increased migratory, invasive, and dissemination properties and chemoresistance of cancer cells. E-cadherin, the prototype of the cadherin superfamily, is a key component of cadherin–catenin–cytoskeleton complexes and maintains the epithelial phenotype, contrasting with the metastatic dissemination and invasiveness of epithelial cells [32]. The loss of this adhesion protein is relatively common in cancers of epithelial origin and correlates with poor prognosis in PDAC [33]. At the protein level, the effect of NAA treatment on E-cadherin expression was analyzed by Western blot analysis. Notably, treatment with the higher concentration of NAA led to a marked increase in E-cadherin levels (Figure 4B). In contrast, the lower dose (4 mM) appeared to have no effect on E-cadherin expression, indicating that this concentration is insufficient to induce a detectable change in protein levels. Confocal microscopy images of BxPC-3 cells treated with NAA 8 mM for 72 h confirmed these findings, showing an increase in the fluorescence of the treated cells, with distinct membrane localization (Figure 4C). Specifically, quantitative analysis revealed an approximately 80% increase in relative fluorescence intensity in NAA-treated cells compared to the untreated condition (Figure 4D).

Taken together, these results led us to conclude that NAA may mitigate the migration of pancreatic cancer cells by regulating E-cadherin expression.

### 3.4. NAA Downregulates the Expression of Stemness Markers

The downregulation of both CD133 and CD184 (CXCR4) stemness markers is the gold standard for exploring the benefits of novel therapeutic strategies aimed at impairing the aggressive, self-renewing, and metastatic properties of migrating cancer stem cells (miCSCs) in PDAC [34]. Because the subpopulation of CD133+/CXCR4+ cancer stem cells was identified as responsible for metastatic dissemination in PDAC, depleting this specific cancer stem cell pool could virtually abolish the metastatic phenotype of pancreatic tumors.

**Figure 5:**
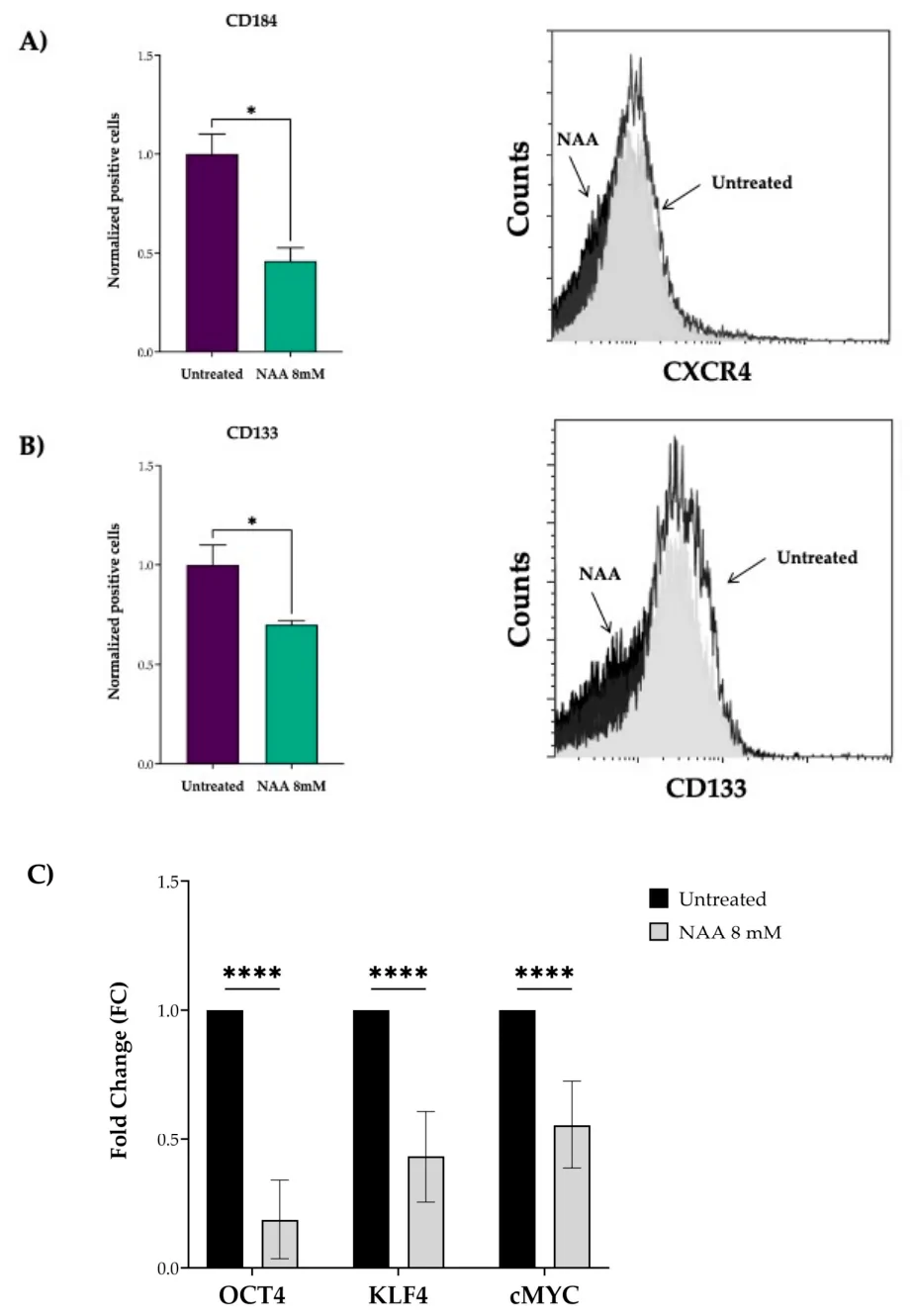
NAA treatment downregulates stemness markers in BxPC-3 cells. Flow cytometry analysis of cancer stem cell surface markers following treatment with 8 mM NAA for 72 h. (**A**) Quantification of normalized positive cells (left) and representative histogram overlay (right) for CD184 (CXCR4) expression. (**B**) Quantification of normalized positive cells (left) and representative histogram overlay (right) for CD133 expression. Bar graph data are presented as mean ± SEM of three independent experiments. Statistical significance was determined by unpaired *t*-test; * *p* ≤ 0.05. (**C**) Transcript expression of OCT4, KLF4 and c-MYC in BxPC-3 cells treated with 8 mM NAA for 72 h. Expression values are presented as fold change (FC) relative to untreated cells. Data are shown as the mean ± SD from three independent experiments. Statistical significance was determined by unpaired *t*-test; **** *p* ≤ 0.0001.

With this aim, the effect of the 72 h 8 mM NAA treatment on the pancreatic stem cell population was evaluated. The small metabolite reduced stemness features by decreasing the percentage of CD133- and CD184-positive BxPC-3 cells, as shown in Figure 5A,B.

Specifically, NAA treatment halved the CD184+ subpopulation of BxPC-3 compared to untreated cells (Figure 5A), while the CD133-positive cells displayed a decrease of roughly 30% (Figure 5B). By acting on two different stemness markers, NAA affected the mesenchymal and stemness phenotype of PDAC cells, inhibiting different signal pathways. In particular, the synergistic loss of both CD133 and CD184 prevents cells from switching to a mesenchymal and migratory phenotype.

Consequently, the cells revert to epithelial characteristics and lose the ability to invade blood vessels. Interestingly, these data highlighted the role of NAA in the regulation of differentiation processes, which was often linked to poor prognosis and metastasis.

In addition to the expression of surface stemness markers, the effect of NAA treatment on stemness attenuation was evaluated by analyzing the expression of key genes involved in the acquisition of stemness features. The transcript levels of *OCT4*, *KLF4*, and c-*MYC* were determined using qRT-PCR. As expected, exposure of BxPC-3 cells to 8 mM NAA for 72 h significantly downregulated these genes (Figure 5C), supporting the impairment of the stem cell-like phenotype.

### 3.5. NAA Treatment Alters Spheroid Assembly, Morphology and Viability

As is well known, CD133-positive cells isolated from several cancer types can give rise to long-term spheroids and xenograft tumors in immunodeficient mice due to their capability for self-renewal and high proliferation [35]. Thus, we evaluated whether the NAA-dependent downregulation of stemness markers, in particular the CD133 antigen, could affect the spheroid formation competence of BxPC-3 cells.

**Figure 6:**
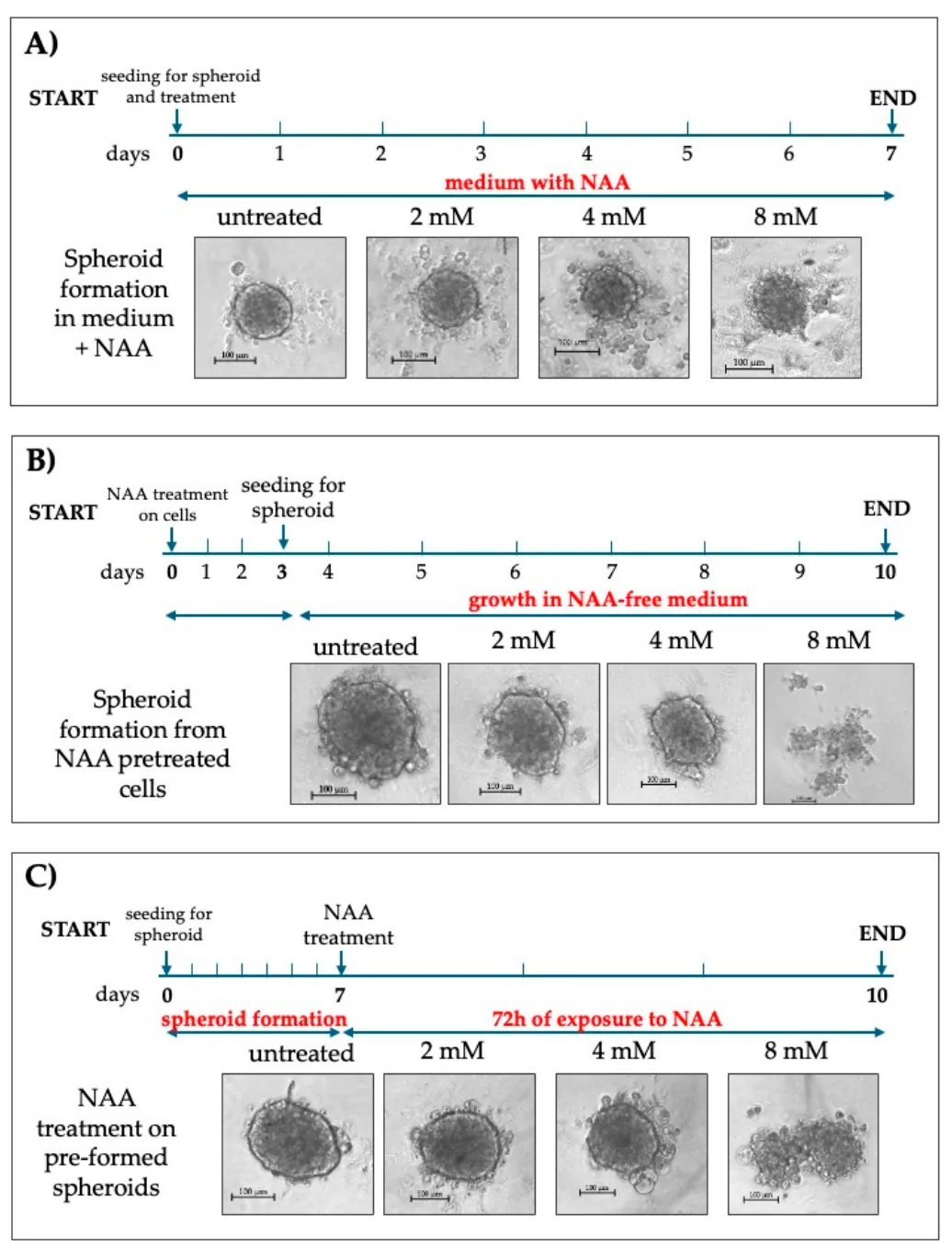
Effects of NAA on BxPC-3 spheroid formation and integrity. Different experimental procedures used to evaluate the effects of NAA on spheroids, together with representative bright-field microscopy images. (**A**) BxPC-3 cells were seeded for spheroid formation and cultured for 7 days in medium containing NAA at the indicated concentrations (2, 4, or 8 mM). (**B**) Cells were pretreated with NAA for 3 days, seeded for spheroid formation, and subsequently cultured for 7 days in NAA-free medium. (**C**) Spheroids were allowed to form for 7 days and were then exposed to NAA at the indicated concentrations (2, 4, or 8 mM) for 72 h. Untreated spheroids were used as controls. Scale bars, 100 μm.

The effect of NAA treatment on spheroid models was evident, perturbing the assembly capacity, shape and viability of the BxPC-3 spheroids. When the NAA treatment was performed simultaneously with cell seeding for spheroid formation, interference in 3D cluster organization was evident even at the lowest tested concentration. As shown in Figure 6A, the boundary of spheroids was irregular at just 2 mM, while unassembled structures appeared at 4 and 8 mM, demonstrating that BxPC-3 cells lost their self-aggregation ability to cluster into spherical, organized aggregates in the presence of NAA. Similarly, when BxPC-3 cells were pretreated with increasing concentrations of NAA before seeding, the morphology of spheroids was altered (Figure 6B). The 3D cluster shape was not perfectly circular, and the overall size was reduced at 2 and 4 mM NAA. At an 8 mM NAA concentration, disaggregated cells were clear, suggesting that the treatment significantly impairs spheroid formation.

Finally, the effect of NAA on pre-formed spheroids was evaluated by treating the 3D models for 72 h with increasing doses of NAA, ranging from 2 mM to 8 mM. As shown in Figure 6C, exposure to NAA degenerated the BxPC-3 spheroids, indicating the impact of the metabolite on both their viability and size.

The effect of NAA treatment on spheroid clusters was also observed in the PANC-1-derived 3D model. After the formation of spheroids, the 72 h exposure to different concentrations of NAA induced a loss of integrity in spheroids at an 8 mM dose (Supplementary Figure S1C). In particular, unlike BxPc-3 spheroids, the PANC-1-derived clusters treated with 8 mM of NAA displayed a more prominent necrotic core, suggesting additional biological effects beyond disaggregation induced by this metabolite.

**Figure 7:**
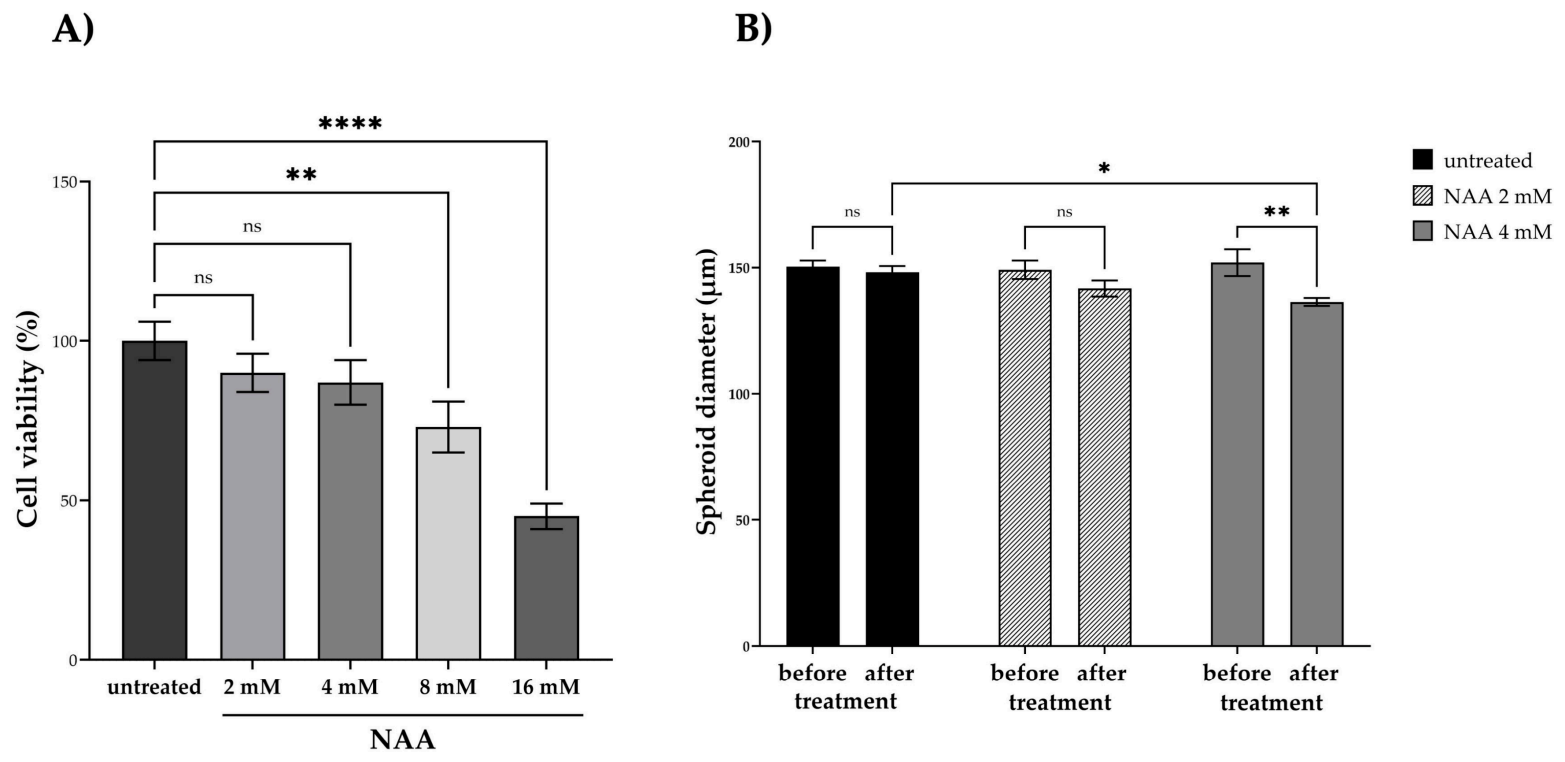
Effects of NAA on the viability and size of BxPC-3 spheroids. (**A**) Cell viability of spheroids treated with increasing concentrations of NAA (2–16 mM), expressed as a percentage of the untreated control. Data are presented as mean ± SD from three independent experiments. Statistical significance, determined by one-way ANOVA, is indicated as follows: ns—not significant; ** *p* ≤ 0.01; **** *p* ≤ 0.0001. (**B**) Spheroid dimensions measured before and after treatment with NAA at 2 or 4 mM; untreated spheroids were used as controls. Data are presented as mean ± SEM of three independent experiments. Statistical significance, determined by two-way ANOVA, is indicated as follows: ns—not significant; * *p* ≤ 0.05; ** *p* ≤ 0.01.

The MTS assay, performed by treating BxPC-3 pre-formed spheroids with NAA in a concentration range of 2–16 mM, confirmed the cytotoxic activity of NAA in spheroids (Figure 7A). Furthermore, the dimensional analysis revealed a significant size reduction in treated clusters compared to untreated ones (Figure 7B) after treatment with 4 mM NAA. Due to its disruptive effect on spheroid assembly, the 8 mM concentration was not included for dimensional analysis.

### 3.6. NAA Downregulates Caveolin-1 Expression and Increases Mitotic Catastrophe After Radiation

Resistance to chemo- and radiotherapy represents one of the major obstacles to overcome to improve the clinical outcomes of PDAC patients. The canonical biological response of pancreatic cancer cells to ionizing radiation is an up-regulation of caveolin-1 (Cav-1) [36]. This protein works like a molecular “shield” that accelerates repair mechanisms of DNA damage [37] induced by irradiation. Thus, the effect of NAA on BxPC-3 sensitivity to radiation was evaluated by combining both treatment strategies.

**Figure 8:**
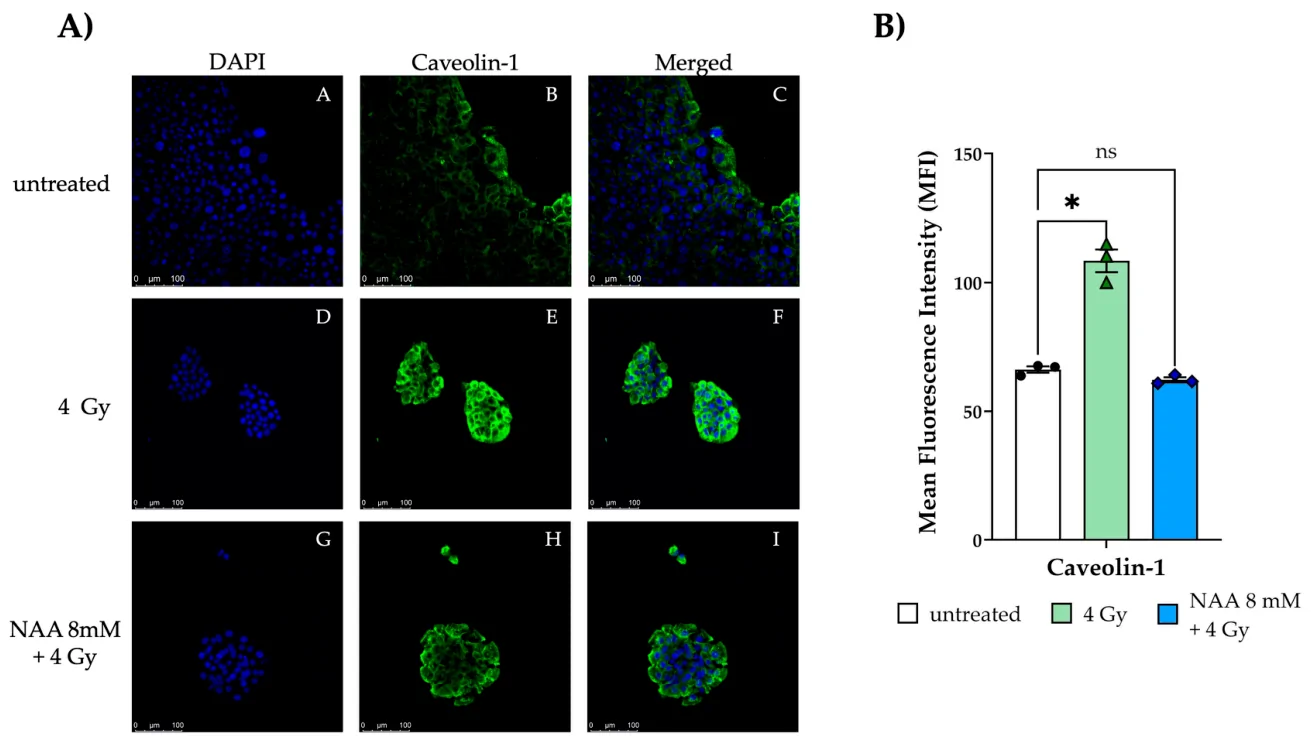
Increase in BxPC-3 sensitivity to irradiation induced by NAA treatment. (**A**) Representative fluorescence microscopy images of caveolin-1 in untreated cells (**A**–**C**), cells exposed to 4 Gy irradiation alone (**D**–**F**) or treated with 8 mM NAA in combination with 4 Gy irradiation (**G**–**I**). Cell nuclei were stained with DAPI (blue), whereas caveolin-1 (green) was detected by immunofluorescence staining. Scale bars, 100 μm. (**B**) Quantification of Mean Fluorescence Intensity (MFI) of caveolin-1 in the three analyzed conditions. The bars represent the mean ± SEM of MFI across three distinct acquisition fields. Statistical significance was evaluated by one-way ANOVA; ns—not significant; * *p* ≤ 0.05.

Confocal microscopy images and quantitative fluorescence analysis revealed that 4 Gy of irradiation significantly increased Cav-1 expression (Figure 8A,B), while the co-treatment with 8 mM of NAA effectively attenuated the Cav-1 upregulation induced by irradiation, driving its expression to baseline levels.

**Figure 9:**
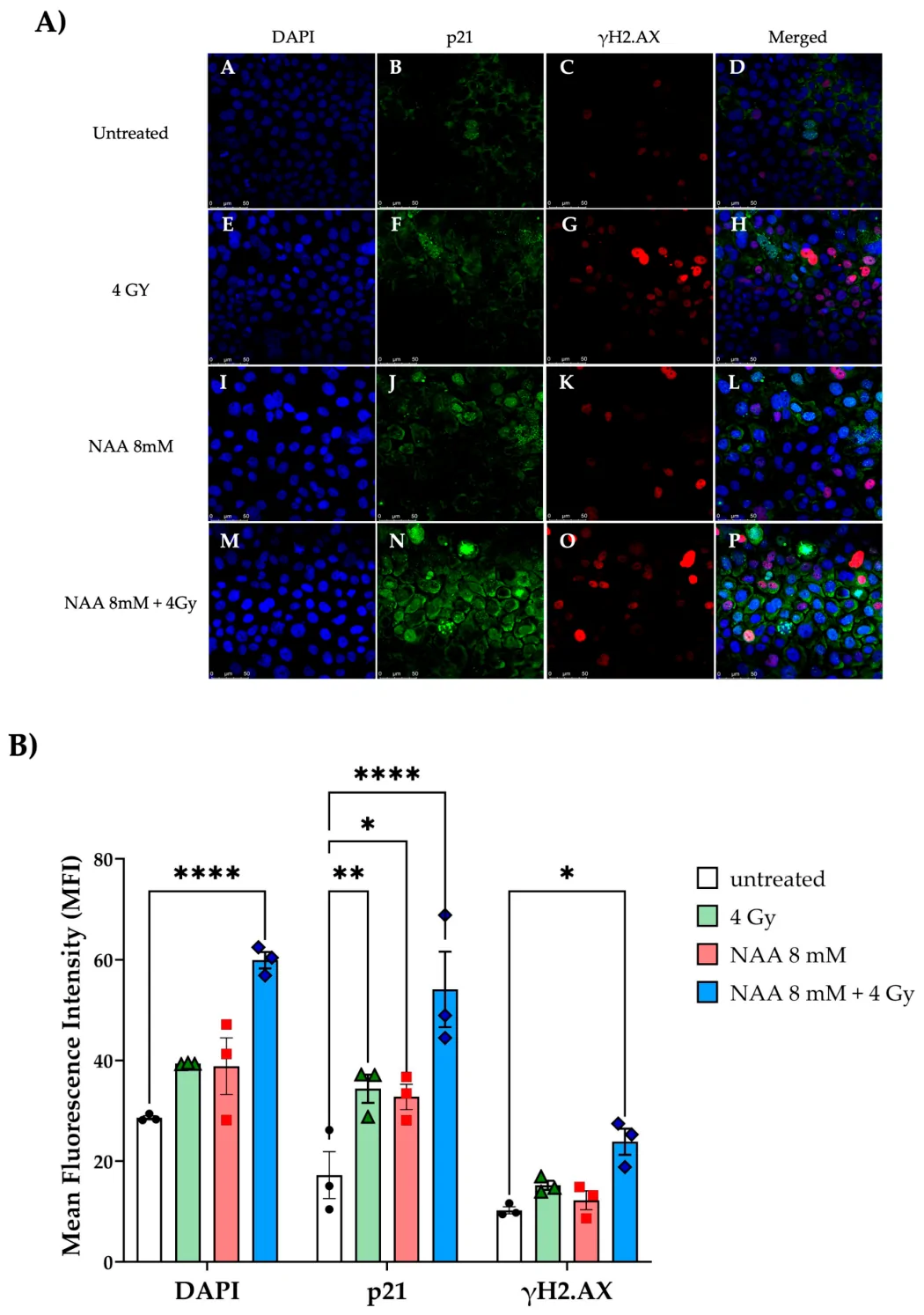
Effect of combination of NAA treatment with irradiation on BxPC-3 cell fate. (**A**) Representative fluorescence microscopy images of p21 and pH2AX expression in untreated BxPC-3 cells (**A**–**D**), those exposed to 4 Gy irradiation alone (**E**–**H**), 8 mM NAA (**I**–**L**), or those treated with 8 mM NAA in combination with 4 Gy irradiation (**M**–**P**). Cell nuclei were stained with DAPI (blue), whereas p21 and pH2AX were detected by immunofluorescence staining, in green and red, respectively. Scale bars, 50 μm. (**B**) Bar graph representing the MFI (mean ± SEM) of nuclei (DAPI), p21 (FITCH-green) and γH2.AX (APC-red) across three distinct acquisition fields. Statistical significance was evaluated by two-way ANOVA; * *p* ≤ 0.05, ** *p* ≤ 0.01, **** *p* ≤ 0.0001.

Furthermore, confocal microscopy analysis at 24 h after irradiation demonstrated that the combination treatment triggered overexpression of γH2.AX and p21, markers of DNA damage and cell cycle arrest respectively (Figure 9A). Quantification confirmed that p21 expression reached its highest level in combination conditions (Figure 9B), pointing to sustained cell cycle arrest driven by NAA. Simultaneously, γH2.AX levels were significantly elevated upon the NAA + 4 Gy treatment (Figure 9B), suggesting a delayed resolution of γH2.AX foci, accumulation of damaged DNA, and an inability to properly divide after replication.

Overall, this therapeutic combined strategy indicates that NAA is not merely cytotoxic; in fact, it may increase sensitivity to irradiation. Thus, NAA-dependent deregulation of Cav-1 may impair tumor defense mechanisms, turning a potentially ineffective radiotherapeutic treatment into a lethal stimulus by taking advantage of the intrinsic mitotic defects of neoplastic cells.

### 3.7. Combination of NAA and Gemcitabine Has Negative Effect on Cell Viability of BxPC-3 Cell Line

**Figure 10:**
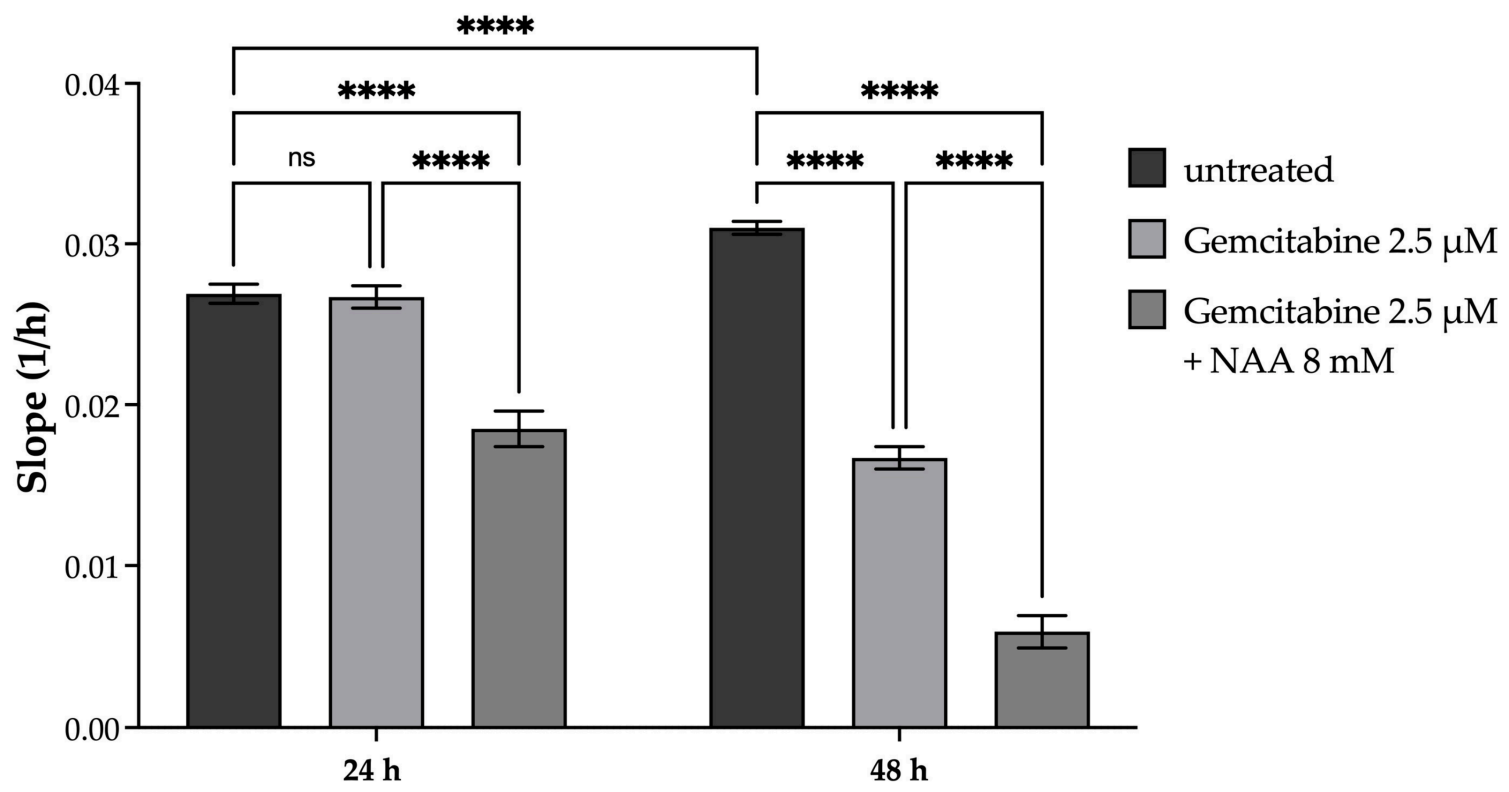
Antiproliferative effect of combined gemcitabine and NAA treatment on BxPC-3 cells. Proliferation rate of BxPC-3 cells treated with different concentrations of NAA, represented by the slope of the normalized CI curves during the 24 and 48 h treatment windows. Bars represent the mean slope ± SD of three experiments. The statistical significance is shown for the indicated comparisons and was calculated using a two-way ANOVA test; ns—not significant; **** *p* ≤ 0.0001.

NAA was evaluated in combination with the antineoplastic drug gemcitabine to investigate whether this metabolite could enhance the anticancer activity of a standard chemotherapeutic agent and overcome drug resistance. Thus, an 8 mM NAA concentration was supplemented with 2.5 μM of gemcitabine. Cell viability was assessed by the xCELLigence system after 24 and 48 h of treatment, and the results are shown in Figure 10. After 24 h, a marked decrease in the viability of BxPC-3 cells was observed after the combined treatment of gemcitabine and NAA compared to that of cells treated with only a chemotherapeutic agent. The antiproliferative effect of the combined treatment was more evident after 48 h of treatment due to the long-term effect of gemcitabine adjuvated by NAA. The significant rise in the slope of untreated cells at the 24 h and 48 h time points confirms that the experimental controls were actively expanding and had not reached a plateau or over-confluence. This excludes growth arrest at 48 h, supporting the conclusion that the pronounced decrease in the proliferation rate is directly dependent on a combination of gemcitabine and NAA.

This last result provides further evidence of the promising anticancer-enhancing effect of the NAA compound on pancreatic cancer cells when used in combination with gemcitabine.

## 4. Discussion

PDAC is a cancer with one of the worst prognoses and survival rates, with limited treatment options and resistance to standard of care (SoC) therapies. Pancreatic tumors are known to be resistant to therapeutic interventions, including targeted therapies, chemotherapy and radiotherapy [38].

In pancreatic cancer, several mechanisms of drug resistance have been suggested, including abnormal gene expression; mutations; deregulation of important signaling pathways (such as NF-κB, Akt and apoptosis pathways); epithelial–mesenchymal transition (EMT); and the presence of stromal cells, highly resistant cells, and stem cells. Each of these mechanisms contributes to drug resistance in pancreatic cancer in different ways and suggests different therapeutic targets [39]. Targeted anticancer drugs are classified into small molecules or macromolecular agents based on the targeted molecules. These include monoclonal antibodies, polypeptides, antibody–drug conjugates, nucleic acid-based therapeutics and cell-based therapies. Small-molecule drugs have a number of practical and pharmacological advantages, including favorable pharmacokinetic properties, oral bioavailability, lower manufacturing and treatment costs, and simplified storage and distribution requirements. Importantly, small-molecule pharmacotherapies are uniquely suited for the treatment of PDAC due to the distinctive genetic and microenvironmental features of this malignancy [40,41].

In the present study, we primarily investigated the efficacy of the endogenous metabolite NAA in 2D and 3D BxPC-3 pancreatic cancer models. To assess the concentration-dependent effects of NAA on malignant phenotypes, BxPC-3 cells were treated with various concentrations of exogenous NAA. Interestingly, while physiological or low millimolar levels of endogenous NAA have been shown to promote metabolic adaptation, treatment with 8 mM of NAA significantly suppressed both cell proliferation and migratory capability in vitro. The analysis of viability parameters of treated cells at an 8 mM concentration of NAA showed a low percentage of apoptotic cells, which suggests a cytostatic rather than a cytotoxic effect exerted by this endogenous small molecule. Additionally, to evaluate whether the observed anti-tumor activity was restricted to this cellular background, selected experiments were also performed in the PDAC KRAS-mutated PANC-1 cell line. NAA significantly impaired PANC-1 proliferation and affected the integrity of PANC-1-derived spheroids at an 8 mM concentration. The similarity of these preliminary results obtained with BxPC-3 and PANC-1 cells suggested that NAA can display its biological activity in a genetically distinct PDAC line.

The epithelial-to-mesenchymal transition and cancer stem cells are intimately associated drivers of therapy resistance in pancreatic cancer. EMT in pancreatic tumor cells leads to the loss of epithelial characteristics and the acquisition of mesenchymal properties responsible for mobility, invasiveness and convergence with stem cell-like properties [42].

Intriguingly, our experimental results showed that NAA treatment modulates phenotypic features associated with EMT and stemness, sustaining a more epithelial-like state in BxPC-3 cells. This observation is supported by increased E-cadherin expression levels, together with a marked reduction in the migration capability of BxPC-3 cells and the downregulation of stemness-associated markers. Consistent with this more epithelial phenotypic shift, NAA demonstrated a decrease in the expression of the cancer stem cell surface markers CD133 and CD184. This effect was further supported by the attenuation in the expression of the stemness-associated genes OCT4, KLF4 and c-MYC. However, due to the predominant epithelial basal phenotype of the BxPC-3 line and our lack of an in-depth analysis of other markers of the epithelial and mesenchymal panel or stemness, these results cannot be interpreted as definitive evidence of the NAA-driven EMT reversal or loss of the cancer-stem-like phenotype.

Tumor spheroids represent a major advance in pancreatic cancer research, bridging the divide between simple cell cultures in Petri dishes and complex living tissues. These models mimic the three-dimensional spatial organization of tumors, cellular interactions and treatment–response gradients around the tumor mass, and they are changing the way oncology drug screening and personalized therapy development are performed [43]. Albeit with some limitations, spheroids can be a predictive model of response to potential pharmacologic treatments. Nevertheless, because the BxPC-3 and PANC-1 spheroid models used for this study were generated exclusively from unique cell lines, they cannot represent the multicellular complexity of the pancreatic tumor and its microenvironment. Notwithstanding these drawbacks, NAA considerably affected BxPC-3 spheroid organization and viability. Moreover, both NAA pretreatment and simultaneous exposure during spheroid formation impaired the assembly of 3D cellular clusters. A comparable loss of spheroid stability was observed in PANC-1-derived spheroids treated with 8 mM of NAA, providing additional evidence that this effect is not line-dependent.

Caveolin-1 is a prognostic biomarker for pancreatic cancer and is essential for pancreatic cell growth, invasion and proliferation in vitro and in vivo. Cav-1 is responsible for resistance to radiation and chemotherapeutics such as gemcitabine and 5-fluorouracil, suggesting that targeting this pathway could improve the therapeutic response [44]. Our data show that NAA treatment in combination with 4 Gy irradiation significantly attenuated the Cav-1 upregulation triggered by ionizing radiation. Moreover, the combined approach increased p21 and resulted in the highest γH2.AX expression 24 h after irradiation. These findings indicate that NAA can interfere with cellular reactions to irradiation and is associated with the persistence of DNA-damage-related responses. However, radiation dose–response clonogenic survival experiments will be required to determine whether NAA may potentially reverse radioresistance in PDAC.

Gemcitabine remains a standard-of-care therapeutic option for pancreatic cancer. However, its clinical efficacy is still limited, and pancreatic ductal adenocarcinoma (PDAC) continues to be associated with one of the shortest overall survival rates among solid malignancies. The promising results observed with the combination of NAA and gemcitabine may therefore provide a rationale for the systematic investigation of novel therapeutic combinations.

Nevertheless, the present findings are mainly based on the BxPC-3 cell line and cannot fully integrate the molecular and phenotypic heterogeneity of pancreatic adenocarcinomas. Further validation in additional PDAC cell lines, as well as therapy-refractory or patient-derived models, will therefore be required to establish the translational relevance of NAA. Certainly, the in vivo models will be fundamental to validate the real potential of a therapy combined with the standard PDAC therapeutic approach.

Overall, these findings provide preliminary preclinical evidence supporting further investigation of NAA as a potential modulator of PDAC cell phenotype and treatment response.

## 5. Conclusions

Current therapies for pancreatic cancer still focus on improving drug delivery and overcoming chemoresistance, with implications that extend well beyond the field of oncology. This study provides in vitro evidence that NAA affects several phenotypes associated with PDAC aggressiveness in BxPC-3 cells. NAA reduced proliferation and migration, promoted G1-phase arrest, increased E-cadherin expression, and decreased the stemness-associated markers CD133 and CXCR4. It also impaired the formation and viability of three-dimensional spheroids. In irradiated cells, NAA prevented caveolin-1 upregulation and was associated with persistent DNA-damage signaling and mitotic catastrophe. In addition, combined exposure to NAA enhanced the antiproliferative effect of gemcitabine. Collectively, these findings support the hypothesis that NAA may act primarily as a therapeutics-sensitizing metabolic modulator rather than as a stand-alone cytotoxic agent. Our study provides a rationale for combining NAA with radiotherapy or standard chemotherapeutics to enhance the clinical efficacy of current PDAC treatment protocols. Further validations in additional PDAC cell lines, both patient-derived and in vivo models, are required to define the pharmacological feasibility and therapeutic relevance of NAA in combined regimens with radiotherapy and chemotherapy.

## Figures and Tables

**Table 1:** Primer sequences used for qRT-PCR experiments.

Gene	Primer Sequence 5’ → 3’
**CDKN1A (p21)**	CGTTCACAGGTGTTTCTGC
	CGGTGACAAAGTCGAAGTTC
**CDKN1B (p27)**	ATCACAAACCCCTAGAGGGCA
	GGAGCCCCAATTAAAGGCG
**TP53 (p53)**	ACTAAGCGAGCACTGCCCAAC
	CCTCATTCAGCTCTCGGAACATC

## Data Availability

The original contributions presented in this study are included in the article/Supplementary Material. Further inquiries can be directed to the corresponding author.

## Supplementary Materials

The following supporting information can be downloaded at https://www.mdpi.com/article/10.3390/cells15171616/s1: Figure S1 Effect of NAA treatment on PANC-1 cells viability and PANC-1 spheroid integrity; Figure S2 Propidium iodide (PI) histogram of cell-cycle analysis on BxPC-3 cells treated with NAA; Figure S3 Migration curves of BxPC-3 cells treated with 4 and 8 mM NAA.

## References

[1] Park W., Chawla A., O’Reilly E.M. (2021). Pancreatic Cancer: A Review. JAMA—J. Am. Med. Assoc..

[2] Leiphrakpam P.D., Chowdhury S., Zhang M., Bajaj V., Dhir M., Are C. (2025). Trends in the Global Incidence of Pancreatic Cancer and a Brief Review of Its Histologic and Molecular Subtypes. J. Gastrointest. Cancer.

[3] Siegel R.L., Giaquinto A.N., Jemal A. (2024). Cancer Statistics, 2024. CA Cancer J. Clin..

[4] Yu W., Zhou D., Meng F., Wang J., Wang B., Qiang J., Shen L., Wang M., Fang H. (2025). The Global, Regional Burden of Pancreatic Cancer and Its Attributable Risk Factors from 1990 to 2021. BMC Cancer.

[5] Truty M.J., Kendrick M.L., Nagorney D.M., Smoot R.L., Cleary S.P., Graham R.P., Goenka A.H., Hallemeier C.L., Haddock M.G., Harmsen W.S. (2021). Factors Predicting Response, Perioperative Outcomes, and Survival Following Total Neoadjuvant Therapy for Borderline/Locally Advanced Pancreatic Cancer. Ann. Surg..

[6] Conroy T., Pfeiffer P., Vilgrain V., Lamarca A., Seufferlein T., O’Reilly E.M., Hackert T., Golan T., Prager G., Haustermans K. (2023). Pancreatic Cancer: ESMO Clinical Practice Guideline for Diagnosis, Treatment and Follow-Up. Ann. Oncol..

[7] Dusetti N., Bachet J.B., Chanez B., Neuzillet C., de Mestier L., Williet N., Frauhoffer N., Nicolle R., Boilève A., Turpin A. (2025). Medical Management of Pancreatic Cancer: From Personalization to Broadening Treatment Strategies. Cancer Treat. Rev..

[8] Moffett J.R., Arun P., Ariyannur P.S., Namboodiri A.M.A. (2013). N-Acetylaspartate Reductions in Brain Injury: Impact on Post-Injury Neuroenergetics, Lipid Synthesis, and Protein Acetylation. Front. Neuroenerg..

[9] Hofer D.C., Zirkovits G., Pelzmann H.J., Huber K., Pessentheiner A.R., Xia W., Uno K., Miyazaki T., Kon K., Tsuneki H. (2019). N-Acetylaspartate Availability Is Essential for Juvenile Survival on Fat-Free Diet and Determines Metabolic Health. FASEB J..

[10] Appu A.P., Moffett J.R., Arun P., Moran S., Nambiar V., Krishnan J.K.S., Puthillathu N., Namboodiri A.M.A. (2017). Increasing N-Acetylaspartate in the Brain during Postnatal Myelination Does Not Cause the Cns Pathologies of Canavan Disease. Front. Mol. Neurosci..

[11] Moffett J.R., Ross B., Arun P., Madhavarao C.N., Namboodiri A.M.A. (2007). N-Acetylaspartate in the CNS: From Neurodiagnostics to Neurobiology. Prog. Neurobiol..

[12] Surendran S. (2009). Upregulation of N-Acetylaspartic Acid Alters Inflammation, Transcription and Contractile Associated Protein Levels in the Stomach and Smooth Muscle Contractility. Mol. Biol. Rep..

[13] Pessentheiner A.R., Pelzmann H.J., Walenta E., Schweiger M., Groschner L.N., Graier W.F., Kolb D., Uno K., Miyazaki T., Nitta A. (2013). NAT8L (N-Acetyltransferase 8-like) Accelerates Lipid Turnover and Increases Energy Expenditure in Brown Adipocytes. J. Biol. Chem..

[14] Prokesch A., Pelzmann H.J., Pessentheiner A.R., Huber K., Madreiter-Sokolowski C.T., Drougard A., Schittmayer M., Kolb D., Magnes C., Trausinger G. (2016). N-Acetylaspartate Catabolism Determines Cytosolic Acetyl-CoA Levels and Histone Acetylation in Brown Adipocytes. Sci. Rep..

[15] Bogner-Strauss J.G. (2017). N-Acetylaspartate Metabolism Outside the Brain: Lipogenesis, Histone Acetylation, and Cancer. Front. Endocrinol..

[16] Chang C.K., Shih T.T.F., Tien Y.W., Chang M.C., Chang Y.T., Yang S.H., Cheng M.F., Chen B.-B. (2021). Metabolic Alterations in Pancreatic Cancer Detected by In Vivo ^1^H-Mr Spectroscopy: Correlation with Normal Pancreas, Pet Metabolic Activity, Clinical Stages, and Survival Outcome. Diagnostics.

[17] Cantor J.R., Sabatini D.M. (2012). Cancer Cell Metabolism: One Hallmark, Many Faces. Cancer Discov..

[18] Krause N., Wegner A. (2024). N-Acetyl-Aspartate Metabolism at the Interface of Cancer, Immunity, and Neurodegeneration. Curr. Opin. Biotechnol..

[19] Zand B., Previs R.A., Zacharias N.M., Rupaimoole R., Mitamura T., Nagaraja A.S., Guindani M., Dalton H.J., Yang L., Baddour J. (2016). Role of Increased N-Acetylaspartate Levels in Cancer. J. Natl. Cancer Inst..

[20] Weindl D., Cordes T., Battello N., Sapcariu S.C., Dong X., Wegner A., Hiller K. (2016). Bridging the Gap between Non-Targeted Stable Isotope Labeling and Metabolic Flux Analysis. Cancer Metab..

[21] Lou T.F., Sethuraman D., Dospoy P., Srivastva P., Kim H.S., Kim J., Ma X., Chen P.H., Huffman K.E., Frink R.E. (2016). Cancer-Specific Production of n-Acetylaspartate via Nat8l Overexpression in Non-Small Cell Lung Cancer and Its Potential as a Circulating Biomarker. Cancer Prev. Res..

[22] Menga A., Favia M., Spera I., Vegliante M.C., Gissi R., De Grassi A., Laera L., Campanella A., Gerbino A., Carrà G. (2021). N-acetylaspartate Release by Glutaminolytic Ovarian Cancer Cells Sustains Protumoral Macrophages. EMBO Rep..

[23] De Falco P., Lazzarino G., Felice F., Desideri E., Castelli S., Salvatori I., Ciccarone F., Ciriolo M.R. (2023). Hindering NAT8L Expression in Hepatocellular Carcinoma Increases Cytosolic Aspartate Delivery That Fosters Pentose Phosphate Pathway and Purine Biosynthesis Promoting Cell Proliferation. Redox Biol..

[24] Mazzoccoli C., Ruggieri V., Tataranni T., Agriesti F., Laurenzana I., Fratello A., Capitanio N., Piccoli C. (2016). N-Acetylaspartate (NAA) Induces Neuronal Differentiation of SH-SY5Y Neuroblastoma Cell Line and Sensitizes It to Chemotherapeutic Agents. Oncotarget.

[25] Mekala J.R., Kurappalli R.K., Ramalingam P.S., Moparthi N.R. (2021). N-Acetyl L-Aspartate and Triacetin Modulate Tumor Suppressor MicroRNA and Class I and II HDAC Gene Expression Induce Apoptosis in Glioblastoma Cancer Cells In Vitro. Life Sci..

[26] Şener L.T., Albeniz G., Dinç B., Albeniz I. (2017). ICELLigence Real-Time Cell Analysis System for Examining the Cytotoxicity of Drugs to Cancer Cell Lines. Exp. Ther. Med..

[27] Stefanowicz-Hajduk J., Ochocka J.R. (2020). Real-Time Cell Analysis System in Cytotoxicity Applications: Usefulness and Comparison with Tetrazolium Salt Assays. Toxicol. Rep..

[28] Ricci F., Subramanian A., Wade M. (2015). Open Access to High-Content Clonogenic Analysis. J. Biomol. Screen..

[29] Mazzoccoli C., Crispo F., Laurenzana I., Pietrafesa M., Sisinni L., Lerose R., Telesca D., Milella M.R., Liu T., Della Sala G. (2023). Biological Evaluation of [4-(4-Aminophenyl)-1-(4-Fluorophenyl)-1H-Pyrrol-3-Yl](3,4,5-Trimethoxyphenyl)Methanone as Potential Antineoplastic Agent in 2D and 3D Breast Cancer Models. Arch. Pharm..

[30] Lettini G., Sisinni L., Condelli V., Matassa D.S., Simeon V., Maddalena F., Gemei M., Lopes E., Vita G., Del Vecchio L. (2016). TRAP1 Regulates Stemness through Wnt/β-Catenin Pathway in Human Colorectal Carcinoma. Cell Death Differ..

[31] Landriscina M., Laudiero G., Maddalena F., Amoroso M.R., Piscazzi A., Cozzolino F., Monti M., Garbi C., Fersini A., Pucci P. (2010). Mitochondrial Chaperone Trap1 and the Calcium Binding Protein Sorcin Interact and Protect Cells against Apoptosis Induced by Antiblastic Agents. Cancer Res..

[32] Loh C.Y., Chai J.Y., Tang T.F., Wong W.F., Sethi G., Shanmugam M.K., Chong P.P., Looi C.Y. (2019). The E-Cadherin and n-Cadherin Switch in Epithelial-to-Mesenchymal Transition: Signaling, Therapeutic Implications, and Challenges. Cells.

[33] Hong S.M., Li A., Olino K., Wolfgang C.L., Herman J.M., Schulick R.D., Iacobuzio-Donahue C., Hruban R.H., Goggins M. (2011). Loss of E-Cadherin Expression and Outcome among Patients with Resectable Pancreatic Adenocarcinomas. Mod. Pathol..

[34] Hermann P.C., Huber S.L., Herrler T., Aicher A., Ellwart J.W., Guba M., Bruns C.J., Heeschen C. (2007). Distinct Populations of Cancer Stem Cells Determine Tumor Growth and Metastatic Activity in Human Pancreatic Cancer. Cell Stem Cell.

[35] Liou G.Y. (2019). CD133 as a Regulator of Cancer Metastasis through the Cancer Stem Cells. Int. J. Biochem. Cell Biol..

[36] Hehlgans S., Eke I., Storch K., Haase M., Baretton G.B., Cordes N. (2009). Caveolin-1 Mediated Radioresistance of 3D Grown Pancreatic Cancer Cells. Radiother. Oncol..

[37] Zhu H., Yue J., Pan Z., Wu H., Cheng Y., Lu H., Ren X., Yao M., Shen Z., Yang J.M. (2010). Involvement of Caveolin-1 in Repair of DNA Damage through Both Homologous Recombination and Non-Homologous End Joining. PLoS ONE.

[38] Espona-Fiedler M., Patthey C., Lindblad S., Sarró I., Öhlund D. (2024). Overcoming Therapy Resistance in Pancreatic Cancer: New Insights and Future Directions. Biochem. Pharmacol..

[39] Pokora B., Pokora K., Fichna J. (2026). Pancreatic Cancer: Emerging Small Molecule Pharmacotherapies. Expert Opin. Pharmacother..

[40] Long J., Zhang Y., Yu X., Yang J., Lebrun D.G., Chen C., Yao Q., Li M. (2011). Overcoming Drug Resistance in Pancreatic Cancer. Expert Opin. Ther. Targets.

[41] Zhong L., Li Y., Xiong L., Wang W., Wu M., Yuan T., Yang W., Tian C., Miao Z., Wang T. (2021). Small Molecules in Targeted Cancer Therapy: Advances, Challenges, and Future Perspectives. Signal Transduct. Target. Ther..

[42] Zhou P., Li B., Liu F., Zhang M., Wang Q., Liu Y., Yao Y., Li D. (2017). The Epithelial to Mesenchymal Transition (EMT) and Cancer Stem Cells: Implication for Treatment Resistance in Pancreatic Cancer. Mol. Cancer.

[43] Jamshidi N., Jamshidi N., Chahardehi A.M., Shams E., Chaleshi V. (2025). A Promising Breakthrough in Pancreatic Cancer Research: The Potential of Spheroids as 3D Models. BioImpacts.

[44] Chatterjee M., Ben-Josef E., Thomas D.G., Morgan M.A., Zalupski M.M., Khan G., Andrew Robinson C., Griffith K.A., Chen C.S., Ludwig T. (2015). Caveolin-1 Is Associated with Tumor Progression and Confers a Multi-Modality Resistance Phenotype in Pancreatic Cancer. Sci. Rep..

