# Supplementary material for: N-Acetyl Aspartic Acid (NAA) Attenuates Stemness and Epithelial–Mesenchymal Transition and Enhances Radio- and Chemo-Sensitivity in Pancreatic Ductal Adenocarcinoma
Source: Cells. 2026 Sep 5;15(17):1616. doi: 10.3390/cells15171616 (PMC13565298; doi:10.3390/cells15171616)
Supplement: Supplementary file 1 [file cells-15-01616-s001.zip › cells-4474987-supplementary.pdf]

**Supplementary Figure S1**

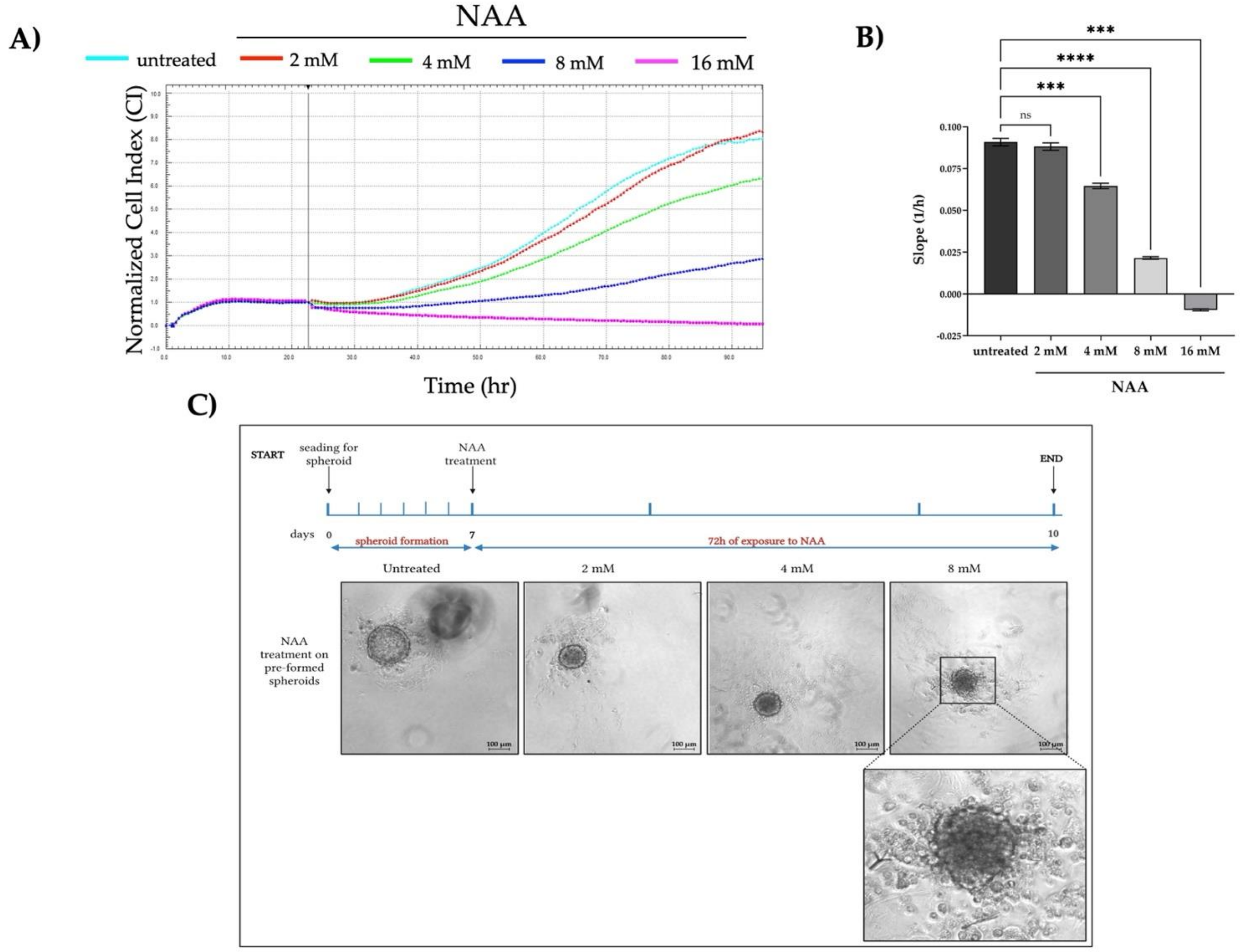


**Supplementary Figure S1. Effect of NAA treatment on PANC-1 cells viability and PANC-1 spheroid integrity. A)** Representative proliferation profile of PANC-1 cells treated with NAA at different concentrations (2, 4, 8, or 16 mM) for 72 h. The cell proliferation assay was performed using the xCELLigence system. Cell index (CI) values were normalized at time of treatment. **B)** Proliferation rate of PANC-1 cells, under NAA treatment at different concentrations, represented by the slope (mean ± SD) of the normalized CI curves during 72 h treatment window. Statistica significance is shown for the indicated comparisons and was determined by one-way ANOVA; ***$p < 0.001$; ****$p < 0.0001$. **C)** PANC-1 cells were seeded to allow spheroid formation for 7 days. The formed spheroids were exposed to NAA treatment for 72h at the indicated concentrations (2, 4, or 8 mM). Untreated spheroids were used as controls.

## Supplementary Figure S2

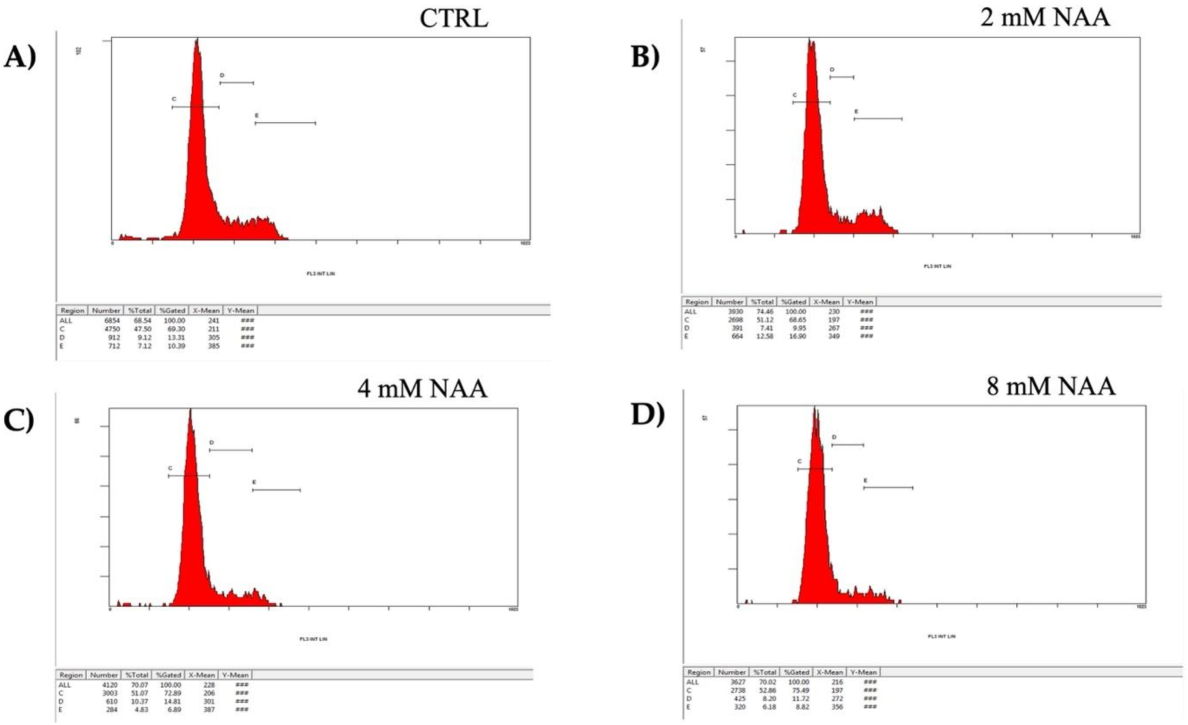


**Supplementary Figure S2. Propidium iodide (PI) histogram of cell-cycle analysis on BxPC-3 cells treated with NAA.** Representative histograms of one cell cycle experiments on BxPC-3 untreated **(A)** or treated with 2 mM **(B)**, 4 mM **(C)** and 8 mM **(D)** NAA for 72 h. The PI histogram display DNA content (x axis, PI fluorescence) versus cell counts (y axis).

## Supplementary Figure S3

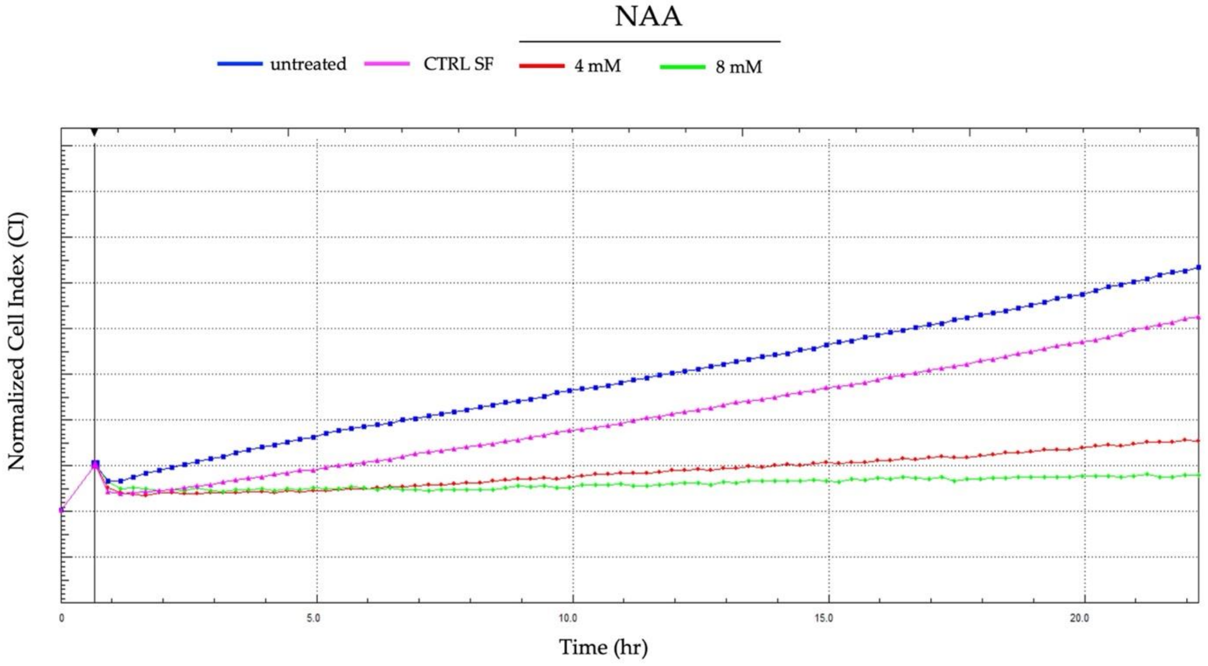


**Supplementary Figure S3 Migration curves of BxPC-3 cells treated with 4 and 8 mM NAA.** Representative migration profile of BxPC-3 cells treated with NAA at different concentrations for 72 h. The migration assay was performed using the xCELLigence system. Cell index (CI) values were normalized at start of experiment.
